# Nuclear peripheral chromatin-lamin B1 interaction is required for global integrity of chromatin architecture and dynamics in human cells

**DOI:** 10.1007/s13238-020-00794-8

**Published:** 2020-11-05

**Authors:** Lei Chang, Mengfan Li, Shipeng Shao, Chen Li, Shanshan Ai, Boxin Xue, Yingping Hou, Yiwen Zhang, Ruifeng Li, Xiaoying Fan, Aibin He, Cheng Li, Yujie Sun

**Affiliations:** 1 State Key Laboratory of Membrane Biology, School of Life Sciences, and Biomedical Pioneering Innovation Center (BIOPIC), Peking University, Beijing, 100871 China; 2 Peking-Tsinghua Center for Life Sciences, Academy for Advanced Interdisciplinary Studies, Peking University, Beijing, 100871 China; 3 Center for Bioinformatics, School of Life Sciences, Peking University, Beijing, 100871 China; 4 Institute of Molecular Medicine, Beijing Key Laboratory of Cardiometabolic Molecular Medicine, Peking University, Beijing, 100871 China; 5 Center for Statistical Science, Peking University, Beijing, 100871 China; 6 Bioland Laboratory (Guangzhou Regenerative Medicine and Health Guangdong Laboratory), Guangzhou, 510530 China

**Keywords:** lamin B1, 3D genome, Hi-C, chromatin dynamics, chromosome territories, A/B compartments, live-cell imaging, super-resolution imaging

## Abstract

The eukaryotic genome is folded into higher-order conformation accompanied with constrained dynamics for coordinated genome functions. However, the molecular machinery underlying these hierarchically organized three-dimensional (3D) chromatin architecture and dynamics remains poorly understood. Here by combining imaging and sequencing, we studied the role of lamin B1 in chromatin architecture and dynamics. We found that lamin B1 depletion leads to detachment of lamina-associated domains (LADs) from the nuclear periphery accompanied with global chromatin redistribution and decompaction. Consequently, the inter-chromosomal as well as inter-compartment interactions are increased, but the structure of topologically associating domains (TADs) is not affected. Using live-cell genomic loci tracking, we further proved that depletion of lamin B1 leads to increased chromatin dynamics, owing to chromatin decompaction and redistribution toward nucleoplasm. Taken together, our data suggest that lamin B1 and chromatin interactions at the nuclear periphery promote LAD maintenance, chromatin compaction, genomic compartmentalization into chromosome territories and A/B compartments and confine chromatin dynamics, supporting their crucial roles in chromatin higher-order structure and chromatin dynamics.

**Electronic supplementary material:**

The online version of this article (10.1007/s13238-020-00794-8) contains supplementary material, which is available to authorized users.

## Introduction

Chromatin in the interphase nucleus of eukaryotic cells is highly compartmentalized and structured. Owing to technological breakthroughs in imaging (Boettiger et al., 2016; Wang et al., 2016; Bintu et al., 2018) and sequencing (Dekker et al., 2002; Dostie et al., 2006; Simonis et al., 2006; Fullwood et al., 2009; Lieberman-Aiden et al., 2009; Nagano et al., 2013), chromatin higher-order structure has been increasingly studied over the last decade. Hierarchical chromatin architecture is composed of loops, TADs, active and inactive A/B compartments, and chromosome territories, in increasing scales. A number of architectural proteins and molecular machineries governing chromatin organization and dynamics have been identified. For instance, CTCF (Nora et al., 2017) and cohesin (Haarhuis et al., 2017; Rao et al., 2017; Schwarzer et al., 2017; Wutz et al., 2017) are partly responsible for the formation and maintenance of chromatin loops and TADs. Nevertheless, CTCF does not impact higher-order genomic compartmentalization and cohesin even limits the segregation of A/B compartments (Nuebler et al., 2018). A few studies also revealed other potential protein candidates responsible for global and hierarchical chromatin organization, including HNRNPU (Fan et al., 2018), SAFB (Huo et al., 2020) and TOPORS (Ji et al., 2020). However, the mechanisms that underlie the insulation and distribution of A/B compartments and chromosome territories remain poorly understood.

Microscopy and chromosome conformation capture techniques provide complementary insights into chromatin higher-order structure and subnuclear chromatin spatial distribution. Genomic regions that belong to A-compartments identified by Hi-C are gene-rich, enriched with euchromatin histone markers and transcriptionally active (Lieberman-Aiden et al., 2009; Steensel and Belmont, 2017). Microscopy reveals that transcriptionally active euchromatic loci prefer to localize in nuclear interior (Solovei et al., 2016). On the contrary, B-compartments identified by Hi-C are gene-poor, enriched with heterochromatin markers, and frequently associated with the nuclear lamina (Lieberman-Aiden et al., 2009; Steensel and Belmont, 2017). These findings are concordant with imaging results that transcriptionally inactive heterochromatin is mainly found near the nuclear periphery and nucleoli (Solovei et al., 2016). In addition, chromosome territories also show subnuclear localization preferences, in which gene-rich chromosomes are generally situated towards the interior and gene-poor chromosomes towards the periphery of the nucleus (Sun et al., 2000). Such spatial correlations make nuclear lamina a plausible candidate that contributes to the segregation and subnuclear distribution of A/B compartments and chromosome territories (Luperchio et al., 2017; Briand and Collas, 2018; Zheng et al., 2018). Despite these insights, whether lamina proteins are responsible for the segregation and localization of higher-order chromatin structure remains elusive.

Chromatin dynamics is intimately related to the hierarchical chromatin structure and nuclear functions (Shao et al., 2018). Previous studies have shown that the diffusion dynamics of genomic loci are not mere Brownian motion driven by thermal fluctuation but are often convoluted with active processes such as transcription and DNA repair (Chuang et al., 2006; Dimitrova et al., 2008; Cho et al., 2014; Ochiai et al., 2015; Gu et al., 2018). So far, most studies on genomic loci dynamics have been carried out in bacteria (Viollier et al., 2004; Javer et al., 2013) and yeast (Albert et al., 2013; Hajjoul et al., 2013; Kim et al., 2013; Verdaasdonk et al., 2013). In yeast, chromatin dynamics appears to be determined by nuclear constraints. In particular, the telomeres and centromeres are known to be tethered to nuclear envelope, which is suggested to contribute to chromosome territory/segregation (Hubner and Spector, 2010). For mammalian cells, previous studies have shown that genomic loci generally undertake a constrained diffusion process associated with nuclear localization (Chubb et al., 2002). Characterization of histone H2B movements by single-molecule tracking showed reduced displacements at the nuclear periphery, where it presented mobility features like lamin A (Nozaki et al., 2017; Lerner et al., 2020). Bronshtein et al. reported that depletion of lamin A can increase chromatin dynamics in U2OS cells (Bronshtein et al., 2015). They emphasized that chromosomal inter-chain interactions mediated by lamin A throughout the nucleus are critical for maintenance of genome organization but did not focus on the tethering of chromatin by lamin A to the nuclear envelope. Besides, they also found a set of nuclear structural proteins that govern chromatin dynamics, including lamins, BAF, Emerin, CTCF and cohesin (Vivante et al., 2018). In addition to the studies that label specific genomic loci, displaced correlation spectroscopy (DCS) has been used to find that chromatin moves coherently across micron-scale regions for a few seconds (Zidovska et al., 2013). However, it is unclear whether chromatin is tethered to the nuclear envelope in mammalian cells as in yeast, and how this sub-diffusion is related to chromatin higher-order structure.

Nuclear lamina consists of many protein complexes. Among them, lamins are the main component in most mammalian cells and can be classified into A- and B-type lamins. Lamin A and C are the most common A-type lamins and are splice variants of the same gene, while B-type lamins, lamin B1 and B2, are the products of two different genes (Shimi et al., 2008). Lamin B1 mainly localizes at the nuclear periphery, while A-type lamins are also found in the nucleoplasm (Kind et al., 2013; Gesson et al., 2016). DamID of lamin B1 has revealed many nuclear lamina-associated genomic regions named LADs. Typically, a mammalian genome contains 1,100–1,400 LADs, including cell type invariant constitutive LAD (cLAD) and cell type-dependent facultative LAD (fLAD) (Kind et al., 2015). 33% of the genome belongs to cLAD and associates with the nuclear lamina in all cell types. While 38% of the genome belongs to constitutive inter-LAD (ciLAD) and does not associate with the nuclear lamina in any cell types (Meuleman et al., 2013). Interestingly, lamin B1 has structural domains that directly bind to DNA or histone and indirectly interact with chromatin through LEM proteins [LAP2 (lamina-associated polypeptide 2)-emerin-MAN1] (Barton et al., 2015). Proteins in the LEM domain protein family share the ability to bind lamins and tether repressive chromatin at the nuclear periphery (Wagner and Krohne, 2007). Thus, lamin B1 can potentially provide anchors for chromatin to regulate its position, higher-order structure and dynamics. In recent studies, the roles of different lamina proteins in chromatin architecture have been investigated in *Arabidopsis thaliana* (Hu et al., 2019), early *C*. *elegans* embryos (Sawh et al., 2020), *Drosophila* S2 cells (Ulianov et al., 2019) and mouse embryonic stem cells (mESCs) (Zheng et al., 2018), with partial conservation across these different species. In brief, disruption of nuclear lamina proteins can reduce the segregation of A/B compartments and change the localization of partial chromatin regions in different species and cell types, but have different degree of impact on chromatin compaction and transcription. However, relevant studies in human cells are still lacking.

In this study, we hypothesized that chromatin-lamina interactions in human cells function in chromatin higher-order structure and dynamics. We applied a combination of imaging and sequencing techniques to characterize the role of lamin B1 in chromatin architecture and dynamics in human breast tumor cells. We found that lamin B1 is required for attachment of LADs, compaction of chromatin and segregation of chromosome territories and A/B compartments, but does not affect TAD structure. Furthermore, depletion of lamin B1 or disruption of interaction between DNA and lamin B1 can increase genomic loci dynamics, owing to chromosome decompaction and redistribution toward nucleoplasm. Taken together, our data suggested that interactions between lamin B1 and chromatin “grab” chromatin towards the nuclear periphery and might act in a tug-of-war with the inner nuclear matrix which pulls chromatin towards the nucleoplasm, thus coordinate establishing and maintaining proper chromatin higher-order structure and dynamics.

## Results

### Lamin B1 depletion leads to chromatin redistribution and decompaction

To explore the potential role of lamin B1 in nuclear chromatin organization, we first investigated the subnuclear distribution of lamin B1 using stochastic optical reconstruction microscopy (STORM) imaging. Lamin B1 was found to be almost exclusively located at the nuclear periphery (Fig. S1A), in contrast to A-type lamins which were located at both nuclear periphery and nucleoplasm (Bronshtein et al., 2015; Gesson et al., 2016) (Fig. S1B). We then created a *LMNB1* (lamin B1 encoding gene)-knockout (KO) MDA-MB-231 breast cancer cell line using the clustered regularly interspaced short palindromic repeats/ CRISPR-associated (CRISPR/Cas) genome editing tool. Proper knockout of lamin B1 was confirmed by Western blot and immunofluorescence (Figs. S1B and S1C). Importantly, no apparent change of cell cycle was detected in lamin B1-KO cells (Fig. S1D), eliminating the possibility that alterations of nuclear organization are due to biased cell cycle. MDA-MB-231 is a human breast cancer cell line with altered chromosome counts. The karyotyping results showed that the total number of chromosomes did not have a significant difference between wild type (WT) and lamin B1-KO cells (Figs. S1E and S1F).

**Figure 1:**
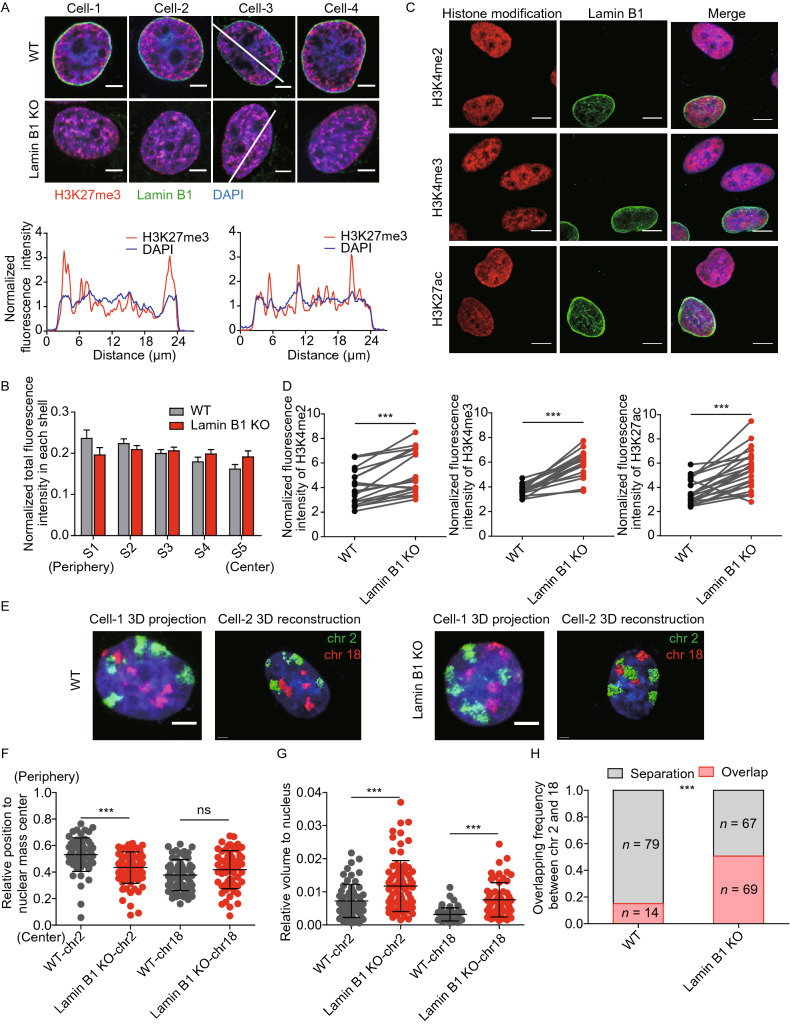
**Lamin B1 regulates chromatin subnuclear localization and global compaction.** (A) Immunostaining of H3K27me3. Red: H3K27me3. Green: lamin B1. Blue: DAPI staining. The 2D sections of nuclei are displayed. Scale bars, 5 µm. Fluorescence intensity along the white line was measured using ImageJ software for H3K27me3 and DAPI channels. (B) Normalized fluorescence intensity of H3K27me3 in each shell in WT (*n* = 35) and lamin B1-KO (*n* = 39) cells. 2 independent experiments. Mean + standard deviation (SD). *P* < 0.05, paired *t* test. (C) Immunostaining of H3K4me2, H3K4me3 and H3K27ac. Red: histone modifications. Green: lamin B1. Blue: DAPI staining. The maximum intensity projections of nuclear Z stacks are displayed. Scale bars, 5 µm. (D) Normalized total fluorescence intensity of immunostaining signals of H3K4me2, H3K4me3 and H3K27ac. Statistical analysis shows increased level of three histone modifications in lamin B1-KO cells. ***P < 0.001, paired *t* test. 2 independent experiments. (E) Representative 3D-projection and 3D-reconstruction chromosome painting images of chromosome 2 and 18. Green: FISH signals of chromosome 2. Red: FISH signals of chromosome 18. Blue: DAPI staining. For 3D-projection images, the maximum intensity projections of nuclear Z stacks are displayed, Scale bars, 5 µm. 3D-reconstruction images are processed in Imaris software, Scale bars, 2 µm. (F) Quantification of the nuclear localization of chromosomes based on their relative distances from the chromosome mass center to the nuclear mass center. This distance is normalized by the cubic root of the nuclear volume. Mean ± SD. ***P < 0.001, Mann-Whitney test. 3 independent experiments. (G) Quantification of the volumes occupied by chromosome 2 and 18 relative to the nuclear volume. Chromosomes in lamin B1-KO cells show significantly larger relative volumes. Mean ± SD. ***P < 0.001, Mann-Whitney test. 3 independent experiments. (H) Quantification of the overlap frequency between chromosome 2 and chromosome 18 territories. The ratio of cells presenting territory interaction between chromosome 2 and chromosome 18 in WT cells is significantly smaller than that in lamin B1-KO cells. ***P < 0.001, Fisher’s exact test. 3 independent experiments

We reasoned that if the anchorage of chromatin to the nuclear periphery is mediated by the interaction with lamin B1, the loss of lamin B1 can lead to changes in distribution and compaction of chromatin in the nucleus. As Zhuang and colleagues have shown that genomic regions with H3K27me3 and H3K4me2 epigenetic modifications have distinct packaging compactness (Boettiger et al., 2016), we imaged histone modifications to evaluate how lamin B1 affects chromatin distribution and compaction. We stained the nuclei with H3K27me3 antibody to visualize the transcriptionally repressive and compact chromatin, and with DAPI to visualize the nuclear profile (Fig. 1A). Both fluorescence intensity along the nuclear diameter and concentric zoning reveals a significant shift in the radial distribution of H3K27me3 from the nuclear periphery towards the interior upon Lamin B1 knockout (Figs. 1A, 1B, S1G and S1H). Meanwhile, we measured H3K4me2, H3K4me3 and H3K27ac histone modifications in 50:50 ratio co-cultured WT and lamin B1-KO cells in the same imaging fields of view (Fig. 1C). We observed that the levels of all three histone modifications that correspond to transcription active were significantly increased in lamin B1-KO cells (Fig. 1D) with no significant difference in nuclear area (Fig. S2A), while the total amount of H3K27me3 remained unchanged (Fig. S2B).

To investigate the effect of lamin B1 on chromatin spatial localization and compaction at the single chromosome level, we performed chromosome painting for chromosomes 2 and 18 using fluorescence *in situ* hybridization (FISH) probes. Chromosomes 2 and 18 were chosen to represent chromosomes that are localized relatively near the nuclear periphery and the nuclear interior, respectively. In lamin B1-KO cells, chromosome 2 became significantly more centrally located while chromosome 18 remained at the nuclear interior (Fig. 1E and 1F). In addition, compared with WT cells, the volume of both chromosomes was significantly increased in lamin B1-KO cells (Fig. 1E and 1G). This expansion of chromosome territories upon lamin B1 depletion is not due to nuclear volume expansion (Fig. S2C). These findings indicate that the nuclear location and volume of individual chromosomes are affected in lamin B1-KO cells. Changes in location and volume of chromosomes may affect the territories between chromosomes. Indeed, along with the redistribution and decompaction of chromatin, more than 50% of lamin B1-KO cells showed overlap between the territories of chromosomes 2 and 18, compared with 15.1% in WT cells (Fig. 1H).

### Lamin B1 depletion results in detachment of LAD from nuclear lamina

**Figure 2:**
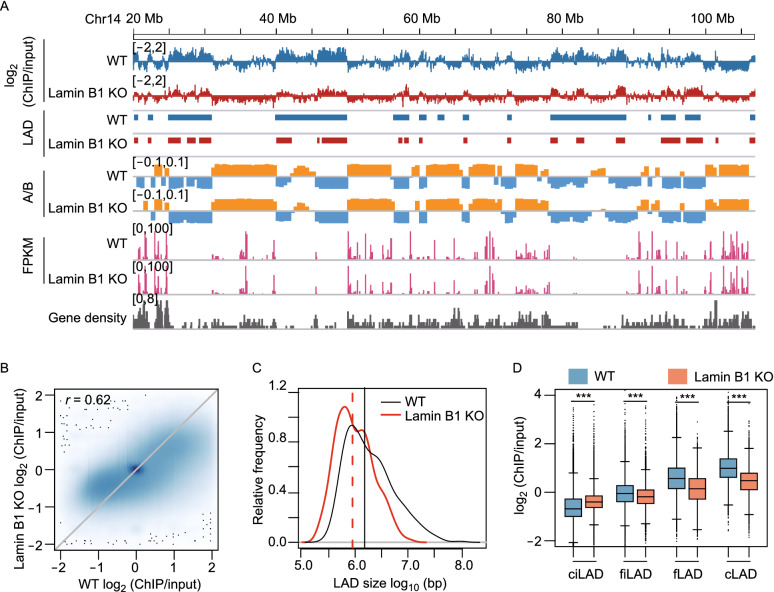
**Lamin B1 is required for the maintenance of LAD.** (A) Genome tracks of chromosome 14 (20–107 Mb) display the log2 (ChIP/input) values of lamin A ChIP-seq data, LADs identified by EDD, A (orange, positive PC1 values) and B (blue, negative PC1 values) compartment, gene expression level (FPKM) in WT and lamin B1-KO cells, and gene density. (B) Scatter plot of log2 (ChIP/input) values in WT and lamin B1-KO cells. 2 independent experiments. (C) Distribution of LAD size in WT and lamin B1-KO cells. 2 independent experiments. (D) log2 (ChIP/input) values for ciLAD, fiLAD, fLAD and cLAD genomic regions in WT and lamin B1-KO cells. ***P < 0.001, unpaired *t*-test

Next, we investigated how lamin B1 KO affects LADs in human breast cancer cells using lamin A chromatin immunoprecipitation (ChIP). Although lamin B1 is almost exclusively located at the nuclear periphery, in contrast to A-type lamins which are located at both nuclear periphery and nucleoplasm (Kind et al., 2013) (Fig. S1B), LADs identified in mammalian cells using DamID fusions with lamin B1, lamin A, emerin, and BAF (Kind and Steensel, 2014), as well as by lamin B1 and lamin A ChIP-seq (Gruenbaum and Foisner, 2015; Briand and Collas, 2020) were found to be highly similar. Consistent with previous studies, higher lamin A ChIP values are correlated with B compartments, repressed gene activity (low FPKM) and gene-poor regions, while lower lamin A ChIP values are correlated with A compartments, active gene activity (high FPKM) and gene-rich regions (Fig. 2A and Table S1). For the whole genome, the lamin A ChIP signals were moderately correlated between WT and lamin B1-KO cells, and the Pearson correlation coefficient was 0.62 (Fig. 2B). Using an enriched domain detector (EDD) peak-calling algorithm (Lund et al., 2014), we identified 376 broad peaks (regarded as LADs) in WT cells, with median length of 1.5 Mb, covering 45% of the genome. In lamin B1-KO cells, the number of LADs increased to 638, while the coverage decreased to 29% of the genome, with median length of 0.9 Mb (Fig. 2C and Table S2). These results indicate that LADs were partly detached from nuclear lamina upon lamin B1 knockout, which resulted in lower LAD coverage and smaller LAD size. We next analyzed the effect of lamin B1 knockout on different classes of LADs. By using LADs identified from public lamin B1 DamID data in human Tig3 fibroblast cells (Guelen et al., 2008), human embryonic stem cells and HT1080 fibrosarcoma cells (Meuleman et al., 2013), and LADs identified from lamin A ChIP-seq data in MDA-MB-231 cells, we divided all genomic regions into 4 different LAD classes based on their lamina-associated behaviors in 4 cell types (Amendola and Steensel, 2015). cLADs are cell-type invariant genomic regions associated with the nuclear lamina in all 4 cell types. ciLADs are genomic regions not associated with the nuclear lamina in all 4 cell types. fLADs are genomic regions associated with the nuclear lamina in MDA-MB-231 cells, but not in any other cell type. Facultative inter-LADs (fiLADs) are genomic regions not associated with the nuclear lamina in MDA-MB-231 cells, but associated with the nuclear lamina in at least one other cell type (Table S3). Compared with WT cells, lamin B1-KO cells had higher lamin A ChIP signals in ciLAD regions, but lower lamin A ChIP signals in cLAD, fLAD and fiLAD regions (Fig. 2D). The latter 3 types of regions are all associated with the nuclear lamina in various degrees in different cell types. These results confirm that loss of lamin B1 contributes to LADs detachment from nuclear lamina globally, consistent with our H3K27me3 imaging results.

### Lamin B1 is required for the segregation of chromosome territories and A/B compartments, but not for TAD insulation

**Figure 3:**
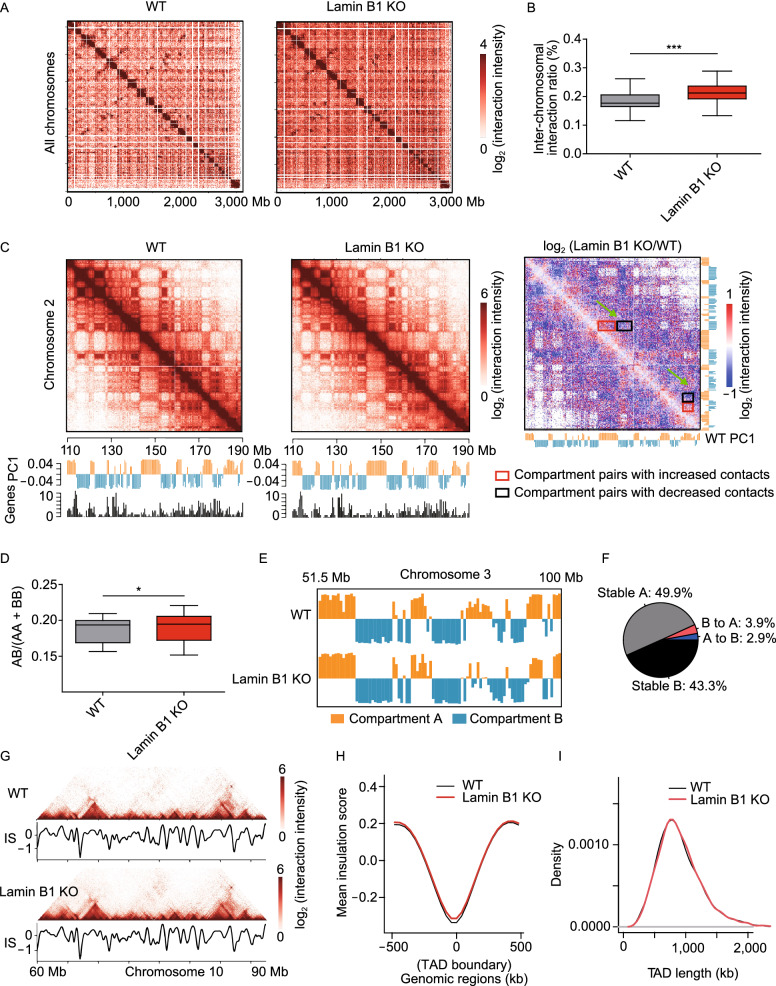
**Lamin B1 depletion reduces the insulation of chromosome territories and A/B compartments.** (A) Normalized Hi-C trans-interaction matrices for the whole chromosomes in WT and lamin B1-KO samples. (B) Trans-interaction ratios of each chromosome in WT and lamin B1-KO cells. For each chromosome, trans-interaction ratio is the percentage of trans-interaction in total interaction of this chromosome. The black lines in the middle of boxplot represent median values, upper and lower ends of boxplot show the upper and lower quartiles, and the whiskers indicate the maximum and minimum values. ***P < 0.001, paired *t*-test. 2 independent experiments. (C) Normalized Hi-C interaction matrices for chromosome 2 (110–190 Mb) in WT and lamin B1-KO cells, and differential matrices of genomic regions between WT and lamin B1-KO cells (resolution: 200 kb). Below the heatmaps are PC1 values and gene density plots. Orange represents compartment A, and blue represents compartment B. High gene density regions correlate with compartment A. (D) Ratios of inter-compartment interactions (AB) and intra-compartment interactions (AA+BB) for each chromosome (X chromosome excluded) in WT and lamin B1-KO cells. *P < 0.05, paired *t*-test. 2 independent experiments. (E) Example of genomic regions transition from A compartment in WT cells to B compartment in lamin B1-KO cells. Compartment A (orange, positive PC1 signal) and compartment B (blue, negative PC1 signal) distribution on chromosome 3 (51.5–100 Mb) in WT and lamin B1-KO cells. (F) Genome-wide summary of genomic regions switching between A/B compartments in WT and lamin B1-KO cells. 2 independent experiments. (G) Example of TAD pattern and insulation score distribution for chromosome 10 (60–90 Mb) in WT and lamin B1-KO cells. (H) Average insulation score distribution around TAD boundaries (±500 kb) in WT and lamin B1-KO cells. 2 independent experiments. (I) Distribution of TAD length in WT and lamin B1-KO cells. 2 independent experiments

These large-scale changes of chromatin prompted us to investigate the role of lamin B1 in genome architecture using *in situ* Hi-C assay (Rao et al., 2014), which provides information about multiscale chromatin interaction maps (Fig. S3A, S3B and Table S4). We first focused on inter-chromosomal interactions. In agreement with the FISH results (Fig. 1E and 1H), Hi-C data showed higher inter-chromosomal interaction frequency between chromosomes 2 and 18 in lamin B1-KO cells (Fig. S3C and S3D), although the interaction frequency between different chromosomes is much less than that within the same chromosome (Table S4) as reported in previous studies (Lieberman-Aiden et al., 2009; Maass et al., 2018). The inter-chromosomal interaction ratio of all chromosomes also showed significant increase in lamin B1-KO cells (Fig. 3A and 3B). These results indicate that lamin B1 contributes to the segregation of chromosome territories.

We next explored whether lamin B1-KO affects the organization of A and B compartments, which are defined using the first principal component (PC1) of Hi-C correlation matrices and correspond to different gene densities and transcriptional activities (Lieberman-Aiden et al., 2009) (Fig. 3C). Using super-resolution imaging, Zhuang and colleagues have shown that adjacent A and B compartments are spatially separated from each other (Wang et al., 2016). Here, although the Hi-C contact maps of WT and lamin B1-KO cells displayed similar checkerboard patterns, the differential heatmap showed loss of intra-compartment interactions (interactions between the A-A or B-B compartment pairs) and gain of inter-compartment interactions (interactions between A-B compartment pairs) in lamin B1-KO cells (Fig. 3C). We asked whether this change influences the interactions between specific compartment types by computing the ratio of interaction frequency between different classes of compartments (AB) versus that between the same classes of compartments (AA and BB) for each chromosome (Du et al., 2017). These ratios showed significant increase in lamin B1-KO cells (Fig. 3D), suggesting that depletion of lamin B1 remodels the segregation of different compartment types. Moreover, 2.9% of genomic regions switched from A compartment in WT cells to B compartment in lamin B1-KO cells, while 3.9% of genomic regions exhibited the opposite switching (Fig. 3E and 3F). These percentages of compartment switching upon lamin B1 depletion were higher than those between replicates (Fig. S3E and S3F). These results indicate that lamin B1 contributes to the formation and segregation of different chromosomal compartment types.

Within A/B compartments, chromatin is further packaged in the form of TADs, which are considered as the basic structural units of chromatin and are largely conserved between cell types and across species (Dixon et al., 2012; Nora et al., 2012; Crane et al., 2015). We calculated insulation scores (Crane et al., 2015) for each 40 kb bin of the Hi-C normalized matrix, and the local minima of insulation scores indicated TAD boundaries. The contact maps and insulation scores of an example region on chromosome 10 showed similar TAD patterns in WT and lamin B1-KO cells (Fig. 3G). For the whole genome, insulation scores were highly correlated between WT replicates (Pearson correlation coefficient, *r* = 0.984) or between lamin B1-KO replicates (*r* = 0.987). Correlation between WT and lamin B1-KO samples (*r* = 0.969) was only slightly lower than that between replicates (Fig. S4A). Heatmaps showed that the distribution of insulation scores around TAD boundaries was similar between WT and lamin B1-KO cells (Figs. 3H and S4B).

To investigate whether the TAD locations were changed upon lamin B1 depletion, each TAD boundary in WT cells was paired with the most adjacent TAD boundary in lamin B1-KO cells. We calculated the genomic distance between these paired TAD boundaries and observed that 87% of the TAD boundaries located within the same or adjacent 40 kb bins, and 92.3% of the TAD boundaries shifted by less than two 40 kb bins (Fig. S4C), comparable to these percentages (95% and 95.8%) between WT or lamin B1-KO replicates. The small number of TAD boundary pairs that are neither overlapping nor adjacent were due to random variation in the calculation of insulation scores (Fig. S4D and S4E). As a result, WT and lamin B1-KO cells have almost overlapping TAD length distribution with median length of 840 kb (Fig. 3I). Furthermore, we calculated the TAD score, which is the ratio of intra-TAD interactions to overall intra-chromosome interactions, for each TAD, and found no difference between WT and lamin B1-KO cells (Fig. S4F and S4G), indicating similar TAD compactness for the two samples. Taken together, lamin B1 loss does not affect the organization of TAD structures.

### Lamin B1 depletion changes the subnuclear location preferences of genomic loci and increases chromatin mobility

**Figure 4:**
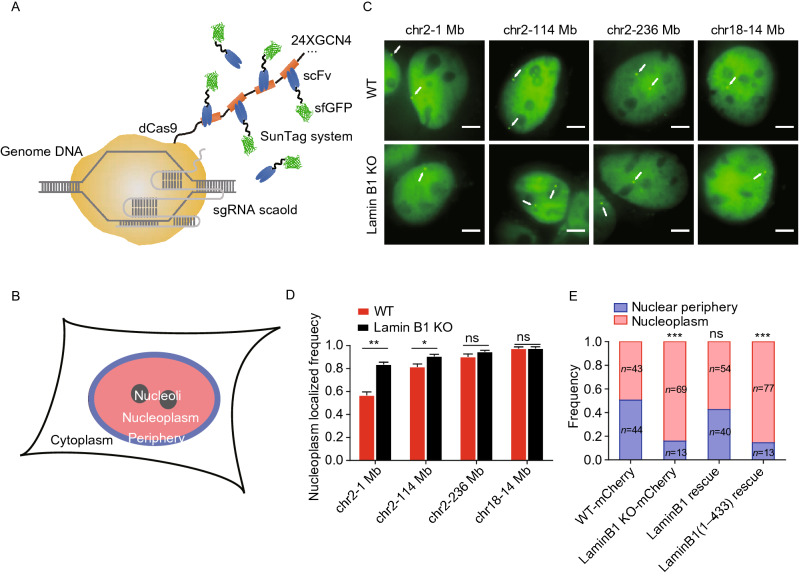
**Lamin B1 depletion changes the location preferences of genomic loci.** (A) Schematic representation of CRISPR-SunTag, a labeling and signal amplification system including dCas9 fused with 24 tandem repeats of GCN4 peptide and a sfGFP-tagged single chain antibody (scFv) for GCN4 peptides. Using dCas9-(GCN4)_24x_ coexpressing with scFv-GCN4-sfGFP at minimal level, a single sgRNA can recruit as many as 24 fluorescent proteins to the target site. (B) Each nucleus is divided into two compartments, nuclear periphery (blue) and nucleoplasm (pink, including nucleoli). (C) CRISPR-SunTag labeling of chr2-1 Mb, chr2-114 Mb, chr2-238 Mb and chr18-14 Mb in WT and lamin B1-KO cells. The white arrows show signals of each loci. Scale bars, 5 µm. (D) The nucleoplasm-localizing frequency of chr2-1 Mb (*n* = 84), chr2-114 Mb (*n* = 117), chr2-238 Mb (*n* = 97) and chr18-14 Mb (*n* = 80) in WT cells, as well as those of chr2-1 Mb (*n* = 88), chr2-114 Mb (*n* = 97), chr2-238 Mb (*n* = 108) and chr18-14 Mb (*n* = 85) in lamin B1-KO cells. **P < 0.01, *P < 0.05, unpaired *t* test. 3 independent experiments. (E) The subnuclear localization changes of 1Mb loci in chromosome 2 in mCherry expressing-WT cells (*n* = 87), mCherry expressing-lamin B1-depleted cells (*n* = 82), lamin B1-rescue cells (*n* = 94) and lamin B1(1-433)-rescue cells (*n* = 90). ***P < 0.001, Fisher’s exact test. 3 independent experiments

Chromatin structures and transcriptional activities are intrinsically associated with its subnuclear location and dynamic motion (Ochiai et al., 2015; Di Pierro et al., 2018; Gu et al., 2018). To further explore the influences of chromatin-lamin B1 interaction on the chromatin, we measured the subnuclear location and dynamics of genomic loci in WT and lamin B1-KO cells. To achieve a high signal-to-noise ratio for precise localization and long-term imaging of genomic loci, we applied CRISPR-SunTag (Tanenbaum et al., 2014), a site-specific chromatin labeling and tracking system, in WT and lamin B1-KO MDA-MB-231 cells (Figs. 4A, S5A, S5B and Table S5). To quantitatively categorize the position of genomic loci, we divided the nuclear space into two regions, i.e. nuclear periphery and nucleoplasm (Fig. 4B and see the “METHODS” section for details). Genomic loci imaging showed that the same genomic loci could localize in different subnuclear regions (Fig. 4C) but did demonstrate location preferences (Fig. 4D). For instance, the 1 Mb genomic locus on chromosome 2 belonging to LAD showed high percentage of nuclear peripheral localization (Fig. 4C and 4D), while the 236 Mb locus on chromosome 2 which is not LAD showed a low percentage of nuclear peripheral localization (Fig. 4C and 4D). In contrast, genomic locus on chromosome 18 which is also non-LAD tended to distribute in the nucleoplasm (Fig. 4C and 4D). In order to avoid measurement artifacts caused by projection from 3D to 2D, we compared the measured distances between loci and nuclear envelope or nucleoli in 2D images and 3D image stacks and obtained similar results (Fig. S5C and S5D). Thus, the overall location of genomic loci in the nucleus coincides with the location of their corresponding chromosome, but different loci on the same chromosome have variable subnuclear localization preferences.

Lamin B1 depletion showed minimal effect on the subnuclear distribution preferences of genomic loci belonging to non-LAD but dramatically altered that of loci near the nuclear periphery (Fig. 4C and 4D). For example, the percentage of the 1 Mb locus on chromosome 2 localized near the nuclear periphery was greatly decreased in lamin B1 depleted cells (Figs. 4C, 4E, S5E and S5F). Lamin B1 may regulate the genomic loci distribution via direct/indirect binding interactions or spatial confinement of accumulative nuclear lamina proteins at the nuclear periphery. To distinguish between these two possibilities, we constructed a plasmid expressing a lamin B1 truncation protein missing the Ig-like domain. The Ig-like domain is a conservative structure in lamin A/C and lamin B1 (Ruan et al., 2012), known as the motif that mediates the direct/indirect interaction between lamins and DNA (Ho and Lammerding, 2012; Luo et al., 2014) (Fig. S5G). In contrast to the exogenous full-length lamin B1 which could rescue the distribution preferences of the 1 Mb locus on chromosome 2 in lamin B1-KO cells, the Ig-like domain-truncated lamin B1 failed to do so (Fig. 4E), even though it could still form the nuclear lamina (Fig. S5H). This result suggests that the tethering between lamin B1 and chromatin is important for the subnuclear position of chromosomes and genomic loci.

**Figure 5:**
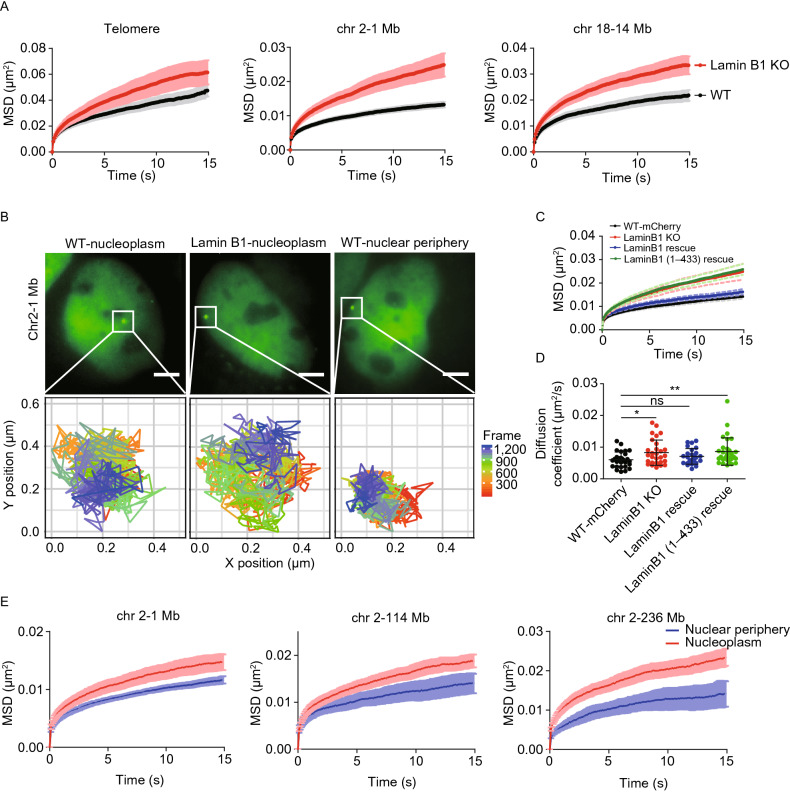
**Loss of chromatin-lamin B1 interaction increases chromatin mobility.** (A) MSD curves of telomeres in WT (*n* = 100) and lamin B1-KO (*n* = 86) cells. MSD curves of 1Mb loci on chromosome 2 in WT (*n* = 27) and lamin B1-KO (*n* = 29) cells. MSD curves of 14 Mb loci on chromosome 18 in WT (*n* = 28) and lamin B1-KO (*n* = 33) cells. Mean ± standard error (SE). 3 independent experiments. (B) The tracking trajectories of labeled 1Mb loci on chromosome 2 in nucleoplasm of WT cells, nucleoplasm of lamin B1-KO cells and nuclear periphery of WT cells. Different colors of trajectories represent time lapse. Scale bars, 5 µm. (C) MSD curves of 1 Mb loci in WT (expressing mCherry, *n* = 29), lamin B1-KO (*n* = 29), lamin B1-rescue (*n* = 25) and lamin B1(1-433)-rescue (*n* = 30) cells. Mean ± SE. 3 independent experiments. (D) The diffusion coefficient of 1Mb loci in WT (expressing mCherry, *n* = 29), lamin B1-KO (*n* = 29), lamin B1-rescue (*n* = 25) and lamin B1(1-433)-rescue (*n* = 30) cells. Mean ± SD. *P < 0.05, *P < 0.01, unpaired *t* test. 3 independent experiments. (E) 3 genomic loci on chromosome 2 are tracked and assigned to nuclear periphery or nucleoplasm compartment, including 1 Mb loci (*n* = 27), 114 Mb loci (*n* = 30) and 236 Mb loci (*n* = 19). Averaged MSD curves of these loci in the two compartments are calculated and displayed as mean ± SE. 3 independent experiments

Next, to investigate how lamin B1 affects chromatin dynamics, three genomic loci consisting of telomeres, a locus localized at 1 Mb on chromosome 2 and a locus at 14 Mb on chromosome 18, were labeled and successively tracked in a short range of time scales (from 0.05 to 120 s) to minimize the artifacts caused by cell deformation, migration or nucleus rotation (Supplementary Video). A tracking package U-track (Jaqaman et al., 2008) was used to extract the trajectories and mean square displacement (MSD) of the loci. The data revealed that depletion of lamin B1 significantly increased chromatin dynamics compared with the slow anomalous diffusion in WT cells of all three loci (Fig. 5A and 5B). Moreover, expressing exogenous full-length lamin B1 in the knockout cell line restored the loci dynamics to the level comparable to WT cells (Fig. 5C and 5D), indicating that lamin B1 restricts chromatin dynamics. However, Ig-like domain-truncated lamin B1 was not able to restore the loci dynamics in lamin B1-KO cells to the WT level as the full-length lamin B1 did (Fig. 5C and 5D), in agreement with the changes of loci localization (Fig. 4E).

To study how chromatin-lamin B1 interaction constrains chromatin dynamics, we first examined the dynamics of the same locus in different subnuclear regions to see whether the genomic loci dynamics are dependent on their locations. Indeed, all three loci on chromosome 2 were much less mobile when located in the nuclear periphery than in the nucleoplasm (Fig. 5B and 5E), suggesting that the dynamics of each locus is primarily influenced by their nuclear spatial environment. Furthermore, we found that the motion of the 1 Mb locus on chromosome 2 in both nuclear periphery and nucleoplasm became more active upon lamin B1 depletion (Fig. S6A). Besides, the 14 Mb locus on chromosome 18, which only had nucleoplasm localization, also showed increased mobility in nucleoplasm in lamin B1-KO cells (Fig. 5A). These results indicate that lamin B1 restrains the mobility of genomic loci in both nuclear periphery and nucleoplasm, not in line with the nuclear lamina distribution of lamin B1. Thus, we speculate that lamin B1 also constrains chromatin dynamics through other ways, especially in the nucleoplasm.

### Chromatin decompaction mediates the effect of lamin B1 depletion on chromatin dynamics

**Figure 6:**
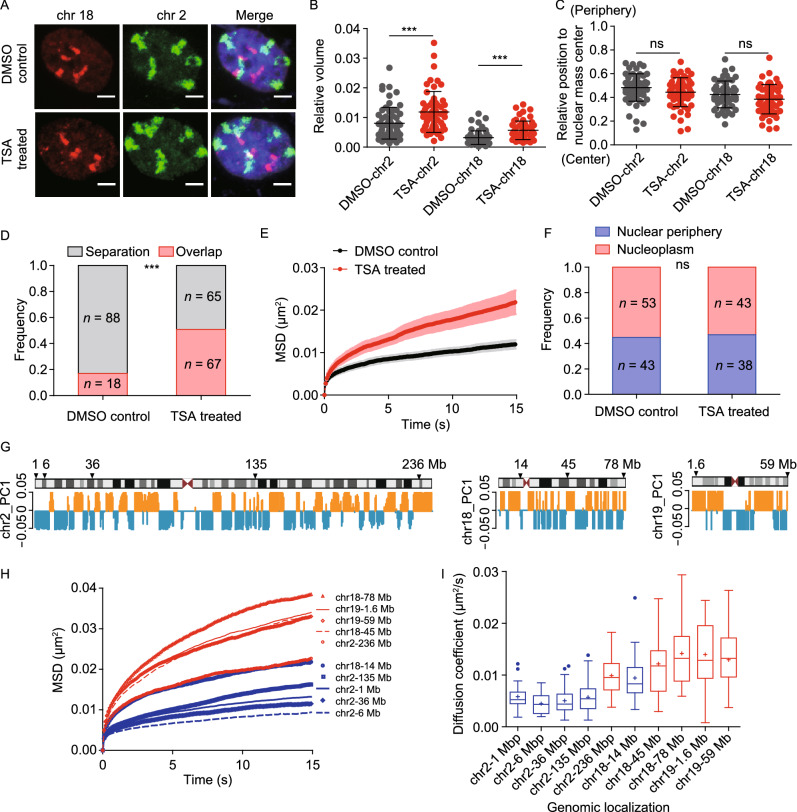
**Global decompaction of chromatin contributes to increased chromatin dynamics and intermingling of chromosome territories.** (A) Representative 3D-projection chromosome painting images of chromosome 2 and 18 in DMSO-treated control cells and TSA-treated cells. Green: FISH signal of chromosome 2. Red: FISH signal of chromosome 18. Blue: DAPI staining. The maximum intensity projections of nuclear Z stacks are displayed. Scale bars, 5 µm. (B) Quantification of the volumes occupied by chromosome 2 and 18 relative to the nuclear volume. Mean ± SD. *** *P* < 0.001, Mann–Whitney test. 3 independent experiments. (C) Quantification of the nuclear localization of chromosomes based on their relative distances from the chromosome mass center to the nuclear mass center. This distance is normalized by the cubic root of the nuclear volume. Mann–Whitney test. 3 independent experiments. (D) Quantification of the overlap frequency between chromosome 2 and chromosome 18 territories. The ratio of cells presenting territory interaction between chromosome 2 and chromosome 18 in control cells (*n* = 106) is significantly smaller than TSA-treated cells (n = 132). *** *P* < 0.001, Fisher’s exact test. 3 independent experiments. (E) MSD curves of 1Mb loci on chromosome 2 in DMSO-treated control cells (*n* = 25) and TSA-treated cells (*n* = 26). Mean ± SE. 3 independent experiments. (F) The spatial localization of 1 Mb loci on chromosome 2 in DMSO-treated control cells (*n* = 96) and TSA-treated cells (*n* = 81). Fisher’s exact test. 3 independent experiments. (G) Schematic representation and PC1 values plots of chromosome 2, chromosome 18 and chromosome 19. Orange represents compartment A, and blue represents compartment B. Arrows indicate the genomic distribution of chosen loci. (H) The averaged MSD curves and (I) The diffusion coefficient distribution of 10 genomic loci on chromosome 2, chromosome 18 and chromosome 19. Red indicates loci belonging to A compartment and blue indicates loci belonging to B compartment. 3 independent experiments

Given the finding that lamin B1 depletion leads to chromatin decompaction (Fig. 1), we next examined whether the increased chromatin dynamics upon loss of lamin B1 was due to chromatin decompaction. We treated WT cells with Trichostatin A (TSA) which can inhibit the histone deacetylase enzyme and lead to genome-wide decondensation of chromatin in both nuclear interior and periphery (Ricci et al., 2015). To confirm the effect of chromatin decompaction on chromosome spatial organization, we applied chromosome painting in TSA-treated cells. We found that the relative volume of chromosomes increased significantly compared with that in control cells (Fig. 6A and 6B), but different from lamin B1 depletion which also altered the position of chromosomes (Fig. 1F), TSA treatment did not change the radial distribution of chromosome territories (Fig. 6A and 6C). The overlap between chromosome 2 and 18 was also consequently increased in TSA-treated cells compared with control cells (Fig. 6A and 6D). We then measured the dynamic mobility of genomic loci and found that TSA treatment indeed promoted the dynamic mobility of genomic loci both near the nuclear periphery and within the nucleoplasm (Figs. 6E and S6B). Importantly, different from lamin B1 depletion, the subnuclear distribution of the loci did not change in TSA-treated cells compared with DMSO-treated control cells (Fig. 6F). These results suggest that chromatin compaction is key for chromatin dynamics.

To further explore the relationship between chromatin dynamics and chromatin compaction state, we chose 10 genomic loci on chromosomes 2, 18 and 19 with 5 in A compartments and 5 in B compartments (Fig. 6G). Tracking of the loci showed that the 5 loci belonging to A compartments (red) were more mobile than the 5 loci in B compartments (blue) (Fig. 6H and 6I), in line with the fact that A compartments are generally less compact than B compartments.

The karyotype and clonal origin of a single lamin B1-KO clone might have an impact on the phenotypes. We verified that the silencing of *LMNB1* also led to increased chromatin mobility for the locus localized at 1 Mb on chromosome 2 in siRNA treated cells (Fig. S7). This result indicated that our findings were not dependent on the lamin B1-KO clone. In our study, chromosomes 2 and 18 were chosen to represent chromosomes that are localized relatively near the nuclear periphery and the nuclear interior, respectively. Previous studies have described that the gene-poor chromosome 18 is located toward the nuclear periphery and the gene-dense chromosome 19 in the nuclear interior (Croft et al., 1999; Kind et al., 2015). The situation, however, is different in cancer cells. Cremer et al. reported that in seven out of eight cancer cell lines, chromosome 18 is located more internally in a higher fraction of nuclei (Cremer et al., 2003). Therefore, we chose chromosome 18 rather than chromosome 19 to represent nuclear interior localized chromosomes in the breast cancer cell line we used. In order to provide more evidence to support that the phenotypes obtained on chromosome 18 can represent nuclear interior localized chromosomes, we also performed experiments on chromosome 19. Chromosome painting results showed that lamin B1 had a similar effect on chromosome 18 and 19 (Fig. S8A–D). The dynamic properties of the genomic locus on chromosome 19 were similar with that on chromosome 18. The 59 Mb locus on chromosome 19, which only had nucleoplasm distribution, also showed increased mobility in nucleoplasm in lamin B1-KO cells (Fig. S8E). Besides, we expressed the Ig-like domain of lamin B1 in lamin B1-KO cells. The results showed that the affected nuclear location, volume and segregation of chromosomes 2 and 19 in lamin B1-KO cells could not be rescued to the extent of expressing full-length lamin B1 in lamin B1-KO cells (Fig. S8A–D). We think that this is because of the abnormal distribution of exogenously expressed Ig-like domain peptide as the nuclear lamina distribution of lamin B1 is mainly determined by their head-to-tail association of dimers (Izumi et al., 2000; Schirmer et al., 2001; Zwerger et al., 2015; Dixon et al., 2017). Thus, expressing the Ig-like domain of lamin B1 alone is not sufficient to rescue the phenotypes independent of its role at the nuclear lamina.

We also performed RNA-seq to study how lamin B1 affect the transcription in the detached genomic regions and globally. The RNA-seq results showed 359 upregulated (fold change > 2, FDR < 0.05) and 493 downregulated genes (fold change < 0.5, FDR < 0.05) in lamin B1 KO cells, with no significant gene ontology (GO) terms for upregulated genes. The downregulated genes are enriched in biosynthetic process and cell migration (Fig. S9A). For the regions where LAD switched to non-LAD upon lamin B1 KO, gene expression was slightly but not significantly decreased (Fig. S9B). For genes in regions where non-LAD switched to LAD, which occupy a small portion of the genome, gene expression level was very low (Fig. S9B and Table S6)

## Discussion

In this study, we found that loss of lamin B1 leads to a fraction of LADs detaching from the nuclear lamina and relocalizing to the nuclear interior. Alongside, we also observed global chromatin decompaction and reduced segregation of chromosome territories as well as A/B compartments upon loss of lamin B1, but the TAD structure was not affected. Several recent studies have also interrogated how lamins affect chromatin architecture in varied species and cell types using different approaches (Zheng et al., 2018; Hu et al., 2019; Ulianov et al., 2019; Sawh et al., 2020). It is important to note that while the main functions of lamina, such as anchoring LADs and segregating A/B compartments, are conserved, there are some differences of lamins in regulating chromatin structure. For instance, lamin *Dm0* knock-down in *Drosophila* S2 cells was found to cause nuclear lamina associated TADs to detach from the lamina and become less tightly packed but the global chromatin compaction was increased (Ulianov et al., 2019). Depletion of *cec-4*, which encodes a chromodomain protein that links methylated H3K9 regions to the lamina in early *C*. *elegans* embryos, caused chromosomes to display smaller radii of gyration and greater compaction (Sawh et al., 2020). In *Arabidopsis thaliana*, lamin-like protein CROWDED NUCLEI 1 (CRWN1) mutant did not cause massive alteration in chromatin accessibility according to ATAC-seq results (Hu et al., 2019). In mESCs, no significant differences were observed in either the volumes or surface areas of chromosomes between lamin triple KO and WT cells, but specific LADs characterized by higher lamin B1 DamID values and lower H3K27me3 exhibit expansion (Zheng et al., 2018). In contrast, in human breast cancer cells in our study, disruption of lamins can lead to global chromatin decompaction, which is in accordance with decondensation of chromosomes 18 and 19 in lamin B1 RNAi DLD-1 cells (Camps et al., 2014). The inconsistent observations could be due to the following reasons. First, cells from different species and differentiated stages were used to study the functions of lamina attachment in regulating chromatin architecture. It is unclear whether lamina have equal functions and chromatin organization has similar dominant driving forces in various biological systems. Of note, lamins are often expressed in a differentiation-dependent manner (Korfali et al., 2012). Second, different protein targets involved in lamina-chromatin association are chosen to study, and even several proteins were disrupted at the same time to decrease the biological redundancy. Previous studies showed diversified mechanisms of chromatin attachment to the nuclear lamina (Towbin et al., 2012; Solovei et al., 2013; Goto et al., 2014; Verboon et al., 2015), and different proteins in the lamina may not function in the same way. Third, different qualitative and quantitative methods were used to judge the same phenotypes. For example, the compaction of chromatin can be evaluated by FISH staining of the entire chromosomes or specific regions, immuno-staining of histone modifications, ATAC-seq, ChIP-seq and modeling. These different qualitative and quantitative approaches may lead to inconsistent conclusions.

Chromatin dynamics have emerged as a new layer of regulation for critical biological processes in eukaryotic nuclei. Despite the active efforts in developing live-cell genomic loci labeling methods during the past few years, little has been done to investigate the principles underlying chromatin dynamics. Here, we applied the CRISPR-SunTag system to label genomic loci in living cells and performed systematic investigation of lamin B1 for its role in chromatin dynamics and subnuclear localization of genomic loci. We found that the motion of genomic loci became more active in lamin B1-depleted cells compared to WT cells, and the chromatin dynamics was dependent on both chromatin compaction and loci location. Furthermore, in WT cells, we consistently observed that genomic loci in less compact compartment A have higher mobility than that in more compact compartment B, suggesting that chromatin compaction is more fundamental than subnuclear location in regulating chromatin dynamics. To our knowledge, this is the first quantitative measurement at the sub-compartment level to unveil the correlation between chromatin dynamics and A/B compartments. Interestingly, this observation is consistent with a recent theoretical work in which a model named MiChroM was proposed for the formation of chromosomal spatial compartments (Pierro et al., 2018). MiChroM defines dynamically associated domains (DADs) in which the motions of genomic loci are correlated. DADs are often found to be aligned with the A/B chromatin-type annotation and another study proposed that the globally increased mobility of genomic loci may drive re-segregation at the chromatin compartment level via modifying MiChroM (Liu et al., 2018). This theoretical work is highly complementary with our experimental data, supporting the important role of chromatin dynamics in establishing higher-order chromatin organization.

How does lamin B1 protein maintain LADs, modulate the physical properties of chromatin, constrain chromatin dynamics, and promote genomic compartmentalization into chromosome territories and A/B compartments? Recently, Falk et al. developed a polymer model of chromosomes to reconstruct chromatin subnuclear localization in inverted and conventional nuclei (Falk et al., 2019). They found that heterochromatin interactions with the lamina are essential for building conventional nuclear architecture. However, regarding the nature of lamin B1-chromatin interaction, it is unclear whether the direct binding (tethering) or the confinement by the lamina meshwork (caging) is the main contributor to the regulation of chromatin structure and dynamics. Here our work has provided two lines of evidence to support the tethering model. First, over-expressing Ig-like domain-truncated lamin B1, which can still form meshwork, was not able to rescue the phenotype of chromatin structure and dynamics caused by loss of lamin B1. Second, overexpressing lamin B1 did not alter the chromatin dynamics. Our results obtained in MDA-MB-231 cells differ with the meshwork caging model proposed by Zheng et al. in mESCs (Zheng et al., 2018), suggesting that cells may tune the functions of lamins along with their differentiation states.

**Figure 7:**
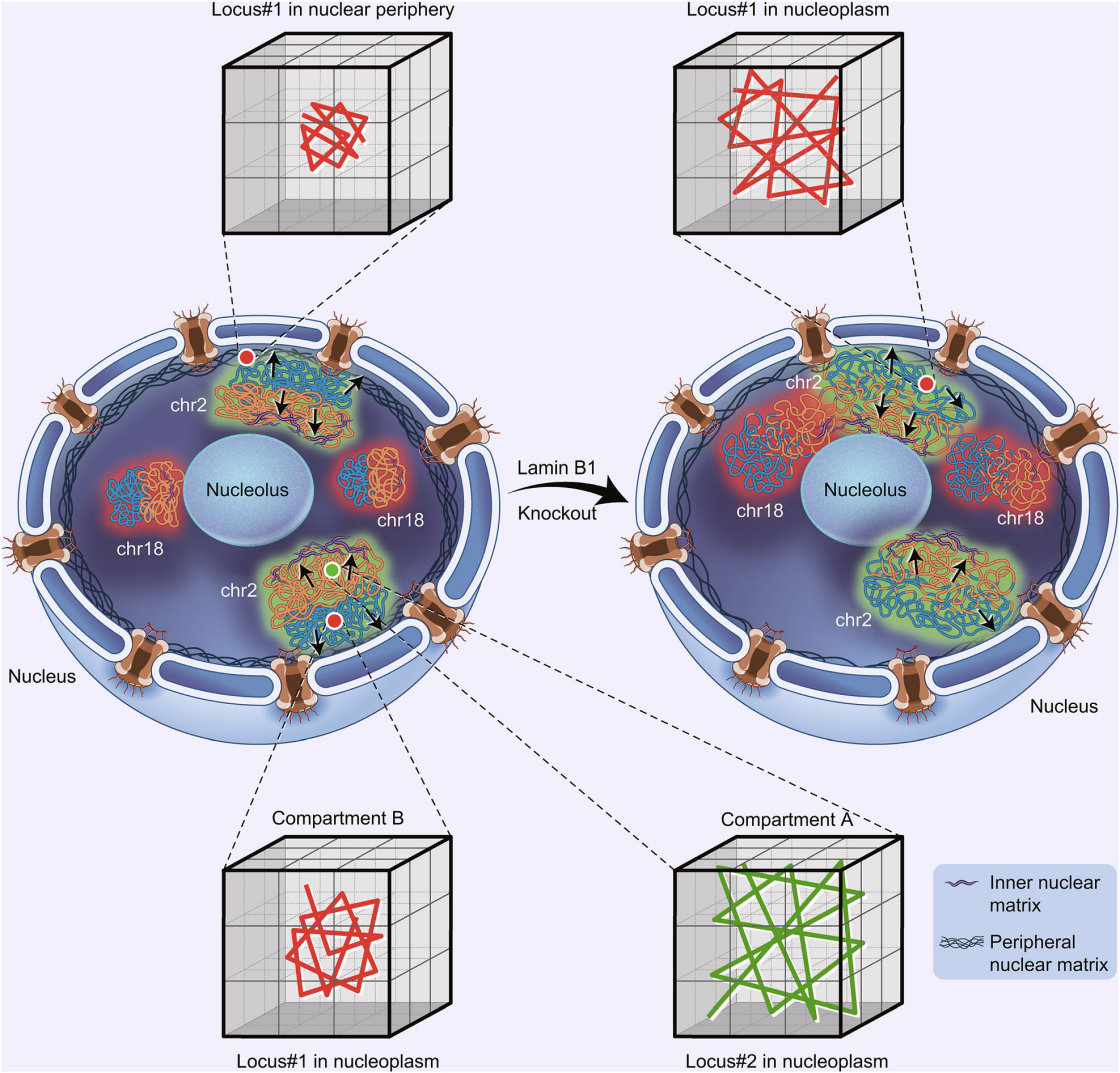
**Model of tug-of-war regulation of 3D chromatin structure by inner and peripheral nuclear matrix proteins.** A model describing lamin B1 tethering chromatin to the nuclear periphery and acting tug-of-war with the nuclear matrix pulling chromatin towards the nuclear interior to coordinate the establishment and maintenance of proper chromatin higher-order structure and dynamics. Loss of lamin B1 in lamina releases a fraction of LADs from the nuclear periphery to nuclear interior, which leads to gain of loosely folded chromatin in nucleoplasm. The change of chromosome compaction state induces expansion of chromosome territories and thus increases the interaction ratio between different chromosomes. Besides, loss of grab on specific LADs reduces the integrity and segregation of chromatin compartments and part of genomic regions switches between A and B compartments. Furthermore, depletion of lamin B1 can increase genomic loci dynamics. The dynamic motion of the same locus in different subnuclear regions demonstrates significant difference, and nuclear periphery-localized loci is much less mobile than the nucleoplasmic-positioned loci. Besides, chromatin compaction is a more fundamental factor affecting chromatin dynamics. Genomic loci in less compact compartment A are of higher mobility than those in more compact compartment B

The interactions between chromatin and lamins can provide a handle for the nuclear lamina to “grab” chromatin. This assumption is supported by our data and other studies (Tajik et al., 2016; Ulianov et al., 2019) that disruption of chromatin-lamin interactions leads to detachment of LADs, nuclear peripheral chromatin shrinkage, and increased interaction with interior chromatin, which consequently result in increased chromatin decompaction and reduced chromatin segregation. Intriguingly, with the lamina pulling chromatin towards the nuclear periphery, it would be tempting to speculate that certain nuclear components could pull chromatin towards the nuclear interior and such tug-of-war mechanism may be essential for establishing and maintaining proper chromatin higher-order structure (Steensel and Belmont, 2017; Li et al., 2020). The nuclear matrix, which is hypothesized to provide a scaffold for chromatin attachment and organize global chromatin structure in the nucleus, is composed of inner and peripheral nuclear matrix. Lamins are main components of peripheral nuclear matrix. Recently, Fan et al. reported that the inner nuclear matrix protein HNRNPU/SAF-A is involved in 3D genome organization (Fan et al., 2018). We compared our lamin B1 data with their HNRNPU/SAF-A data and interestingly found that they contribute to chromatin organization in an opposite manner, implicating some fundamental coordinations between inner and peripheral nuclear matrix in regulation of chromatin structures. For instance, genes enriched in cell adhesion are up-regulated in HNRNPU depleted cells but down-regulated in lamin B1 knockout cells (Fig. S9A). At the A/B compartment level, depletion of HNRNPU and lamin B1 both result in a ~10% transition between A/B compartments. More importantly, in contrast to our findings that depletion of lamin B1 promotes chromatin decompaction and relocalization from nuclear periphery to nucleoplasm, loss of HNRNPU promotes global condensation of chromatin and increases lamina-associated genomic regions. Therefore, these two studies demonstrate that the inner and peripheral nuclear matrix, through anchoring of chromatin in the nucleoplasm and the nuclear envelope respectively, may offer a complementary, tug-of-war regulation of higher-order chromatin organization (Fig. 7).

## Materials and Methods

### Construction of sgRNA expression plasmids and SunTag PiggyBac plasmids

The mining process for repeats was similar as described recently (Ma et al., 2015). Briefly, the human genome sequence was downloaded from the UCSC genome browser (http://genome.ucsc.edu) with undetermined regions “Ns” replaced by randomly generated nucleotides “A”, “T”, “G”, or “C”. Then the sequence was input to the Tandem Repeat Finder bioinformatics tool (http://tandem.bu.edu/trf/trf.html) to identify the tandem repeats. Highly conserved repeats with little mutation, proper repeat unit length and repeat number were selected as candidates for live-cell fluorescent labeling and imaging. sgRNA oligoes targeting each repeat were designed upstream of proto-spacer adjacent motif (PAM) sequence “NGG”. The oligoes of each sgRNA that target the repeat regions on human chromosomes were synthesized by Beijing Ruibo biotech (Beijing, China) with a 4 bp overhang flanking the sense and antisense strands. The sgRNAs targeting lamin B1 gene were designed by the online tool Optimized CRISPR Design (http://crispr.mit.edu) and candidates with the highest score were selected. The sgRNA expression vector for imaging was based on the psgRNA2.0 transient expression plasmid with an A-U flip and stem-loop extension (a gift from Prof. Wensheng Wei, Peking University), containing the ccdB screening gene between two BsmBI sites for inserting guide sequences into the sgRNAs. The sgRNA expression vector for editing was based on plasmid pX330-U6-Chimeric_BB-CBh-hSpCas9 (Addgene Plasmid # 42230), containing two BpiI restriction sites for inserting guide sequences into the sgRNAs. The targeting sgRNA expression plasmids were made by replacing the lethal gene ccdB with annealed oligo using Golden Gate cloning with enzyme BsmBI and T4 ligase (NEB). For the sequence of each sgRNA construct, see Table S5.

In order to construct a stable cell line, the NLS_SV40_-dCas9-3X NLS_SV40_-24X GCN4_-V4_-NLS_SV40_-P2A-BFP fragment was amplified by PCR from plasmid pHRdSV40-NLS-dCas9-24xGCN4_v4-NLS-P2A-BFP-dWPRE (Addgene Plasmid #60910) and then ligated into PiggyBac plasmid pB-TRE3G-BsmBI-EF1α-PuroR-P2A-rtTA by Golden Gate Assembly with enzyme BsmBI and T4 ligase (NEB). The ScFV-sfGFP-GB1-NLS_SV40_ fragment was amplified by PCR from plasmid pHR-scFv-GCN4-sfGFP-GB1-NLS-dWPRE (Addgene Plasmid # 60906) and then ligated into PiggyBac plasmid pB-TRE3G-BsmBI-EF1α-HygroR-P2A-rtTA by Golden Gate Assembly with enzyme BsmBI and T4 ligase (NEB).

### Cell culture, transfection, TSA treatment and RNAi

Human cell line MDA-MB-231 cells were maintained in Dulbecco’s modified Eagle medium with high glucose (Lifetech). The medium contained 10% Fetal bovine serum (FBS) (Lifetech), and 1% of penicillin and streptomycin antibiotics (Lifetech). Cells were maintained at 37 °C and 5% CO_2_ in a humidified incubator. All plasmids were transfected with Chemifect (Beijing Fengrui Biotech, Beijing, China) in accordance with the manufacturer’s protocol. TSA (Sigma-Aldrich) was eluted to 3 mmol/L in DMSO. Cells were treated with 300 nmol/L of TSA solution in complete growth medium for 24 h before imaging experiments and the negative control sample was treated with DMSO. siRNAs targeting lamin B1 (Camps et al., 2014) 5′-GCATTAAAGCAGCGTATC-3′ and a negative control were was synthesized by Shanghai GenePharma Company and RNAi experiments were performed as reported in the manufacturer’s protocol. Briefly, MDA-MB-231 cells were transfected by Lipofectamine RNAiMAX transfection reagent (Thermo Fisher Scientific) in 0 h and 48 h. After the 2nd transfection, cells were left to recover for 24–48 h, and then harvested for downstream experiments and analysis.

### CRISPR-mediated lamin B1 gene knockout

In order to knockout lamin B1 genes, the cells were co-transfected with corresponding sgRNA and Cas9 chimeric plasmid and an empty mCherry expressing plasmid. At 48 h post transfection, cells were subjected to FACS to isolate mCherry positive single cell clone in 96-well plates. After incubation for about a month, genome of each grown clone was extracted and PCR-amplified with lamin B1-specific primer and sent for Sanger sequencing. Three alternative clones were got after Sanger sequencing. However, we could not make sure whether all 4 alleles of lamin B1 had been disrupted. Thus, we did western blot and found that clone#3 had a small amount of lamin B1 expression even though it had the indel, which means it has at least one complete allele. Finally, we did immuno-fluorescent staining of lamin B1 in clone#1 and clone#2, and the images showed that very weak lamin B1 antibody signals appeared in nuclear membrane of clone#2 cells. We chose clone#1 which had no enrichment of lamin B1 antibodies in nuclear membrane to do the following experiments, because we considered that the domain determined lamin B1 subnuclear localization was disrupted in all alleles.

### Construction of the SunTag stable cell line

To construct the stable cell line, MDA-MB-231 cells were spread onto a 6-well plate one day before transfection. On the next day, the cells were transfected with 500 ng pB-TRE3G- NLS_SV40_-dCas9-3X NLS_SV40_-24X GCN4_-V4_-NLS_SV40_-P2A-BFP-PuroR-P2A-rtTA, 500 ng pB-TRE3G-ScFV-sfGFP-GB1-NLS_SV40_-HygroR-P2A-rtTA, and 200 ng pCAG-hyPBase using Chemifect. After 48 h, cells were subjected to hygromycin (200 μg/mL) and puromycin (5 μg/mL) selection. After incubation for two weeks, cells with appropriate expression level of BFP and GFP were selected using FACS. Single cell clones were harvested for imaging a month later.

### Immunofluorescence

Cells were grown on 35 mm glass bottom dish. After the coverage of cells reached 60%–70%, cells were fixed with 4% PFA for 15 min, permeabilized with 0.5% Triton in PBS for 5 min and then blocked in blocking buffer containing 5% BSA and 0.1% Triton for 30 min. The cells were then incubated with primary antibodies in blocking buffer for 1 h at room temperature, washed with PBS three times, and then stained with organic dyes-labeled secondary antibodies in blocking buffer for 1 h at room temperature. The labeled cells were washed again with PBS, then post-fixed with 4% PFA for 10 min and finally stained with DAPI (Invitrogen).

Primary antibodies used in this study were lamin A/C (ab40467, Abcam, dilution 1:200), lamin B1 (sc6216, Santa Cruz, dilution 1:200), H3K4me2 (9725, Cell Signaling Technology, dilution 1:400), H3K4me3 (9751, Cell Signaling Technology, dilution 1:400), H3K27ac (8173, Cell Signaling Technology, dilution 1:100), H3K27me3 (9733, Cell Signaling Technology, dilution 1:400). Secondary antibodies were donkey anti-rabbit Alexa Fluor 555 (A-31572, Thermo Fisher Scientific), donkey anti-rabbit Alexa Fluor 594 (A-21207, Thermo Fisher Scientific), donkey anti-goat Cy5 (705-005-147, Jackson Immuno Research Laboratories, dilution 1:50), donkey anti-mouse Cy3b (715-005-151, Jackson Immuno Research Laboratories, dilution 1:50).

### Optical setup and image acquisition

Briefly, all dynamic tracking experiments in living cells were performed on an Olympus IX83 inverted microscope equipped with a 100× UPlanSApo, N.A. 1.40, oil-immersion phase objective and EMCCD (DU-897U-CS0-#BV). The microscope stage incubation chamber was maintained at 37 °C and 5% CO_2_. A 488 nm laser (2RU-VFL-P-300-488-B1R; MPB) was used to excite the sfGFP fluorophore. The laser power was modulated by anacousto-optic-tunable-filter (AOTF) and the beam width was expanded fivefold and focused at the back focal plane of the objective. The power density at the sample, with epifluorescence illumination, was 10 µW at 488 nm. The microscope was controlled by home-written scripts. Movies of chromatin dynamics in living cells were acquired at 10 Hz. The motions of loci were studied by recording their trajectories in 2D rather than in 3D to increase time resolution and reduce phototoxicity. According to previous study, there is no significant difference in the movement volumes and diffusion coefficient of telomeres between different cell cycle stages in interphase, thus we collected images in interphase without further distinguishing between sub-stages of interphase.

For fixed cell conventional imaging experiments, an UltraVIEW VoX spinning disc microscope (PerkinElmer) or Dragonfly confocal microscope (Andor) was used. STORM imaging of lamin B1 was done on N-STORM (Nikon, Japan)

### Image analysis

All dynamic tracking image stacks were analyzed using MATLAB tracking package “U-track”. Fluorescent puncta were identified in each frame with 2D Gaussian fitting after Fourier low-pass filtering. The coordinates of the fluorescent puncta were determined. Trajectories were created by linking identified puncta to their nearest neighbor within a maximum distance range of 5 camera pixels (800 nm) in the previous frame. Particles with trajectory gap larger than 10 consecutive frames were treated as two particles.

For each trajectory, the mean square displacement (MSD) as a function of time delay was calculated by the following equation:

$$MSD\left( {n\delta t} \right) = \frac{1}{N - 1 - n}\mathop \sum \limits_{j = 1}^{N - 1 - n} \times \left\{ {\left. {\left[ {x\left( {j\delta t + n\delta t} \right) - x\left( {j\delta t} \right)} \right]^{2} + \left[ {y\left( {j\delta t + n\delta t} \right) - y\left( {j\delta t} \right)} \right]^{2} } \right\}} \right.$$

where δt is the time interval between two successive frames, x(t) and y(t) are the coordinates at time t, N is the total number of frames, and n is the number of time intervals. To maximize precision in long-range MSD, intervals smaller than N/10 were used for the calculation.

The analysis of MSD curves was carried out using custom MATLAB scripts. Each individual MSD curve was fitted by least-squares regression to the following model:

$$MSD = Dt^{\alpha }$$

where D is the diffusion coefficient and α is the scaling factor. For each repeat, many trajectories were fitted and grouped. Additionally, every collected cell was inspected carefully, and any cell with slight motion was discarded to eliminate the contamination of such drift in the analysis of loci trajectories.

The immunofluorescence images were acquired on an UltraVIEW VoX spinning disc microscope (PerkinElmer). For each imaging view, z-stacks covering the whole nuclei with a step size of 400 nm were taken for each channel and imaging conditions were kept for different views of one sample. DAPI was stained to represent the nuclear profile. To reduce the photobleaching effect on quantification, the histone modification channel was scanned first. Histone modification quantification was done with ImageJ software. All z sections were projected to one image by the sum slices mode, and the mean intensity and area of each nucleus were measured. The fluorescence background in the cytoplasm was subtracted. The total fluorescence intensity of histone modifications was then normalized by dividing the same number to adjust shown range in y-axis. Each point on the scatter plot represents a cell. About 20 pairs of cells within the same imaging view were analyzed for each independent experiment. Using ImageJ, we measured H3K27me3 profiles across the nucleus diameter of the equatorial focal plane of nuclei of WT and lamin B1-KO cells. Fluorescent intensities were extracted, individual profiles were first delimited by peaks of DAPI fluorescence, then normalized on the average intensity and further aligned to determine the averaged profile. For the spatial distribution analysis of H3K27me3 immunofluorescence images, each nucleus was divided into 5 nuclear shells with equal area form the nuclear periphery to interior using custom MATLAB script. Then the fluorescence intensity in each shell was summed and divided by the total fluorescence intensity of the whole nucleus for normalization. Nuclei from 2 independent experiments were analyzed.

The three-dimensional image analysis was carried out in Imaris (Bitplane) by ImarisCell, a module designed specifically to identify, segment, track, measure and analyze cell, nucleus and vesicles in 3D images. Using “Cell Boundary from Cytoplasm” function, the nucleus was segmented by DAPI channel as the nuclear boundary and the genomic loci were fitted with 3D ellipsoid function as a spot. Then the shortest distance between the spot and the surface was calculated. For chromosome painting image analysis, “Surface” function was used to segment nuclear boundary by DAPI channel and territory boundary of chromosome 2 and chromosome 18/19 by 488 nm and 561 nm channel intensity. The volume and center of mass of nucleus and chromosome territories were output directly. The volume of each nucleus was measured to normalize the volume of chromosome territories. Distance between nuclear center of mass and chromosome territories was normalized by the cubic root of nuclear volume. Considering the copy number of chromosome 2, chromosome 18 and chromosome 19 would be ranged from 4 to 8, depending which cell cycle stage the cell is in. We only selected pairs of nuclei which have the same chromosome copies for chromosome painting analyses.

The threshold for subnuclear position assignment of loci was as follows: 4 pixels’ distance between the locus and nuclear envelope (640 nm), referring to previous publication about LMNB1 LAD FISH analysis (defined there as < 700 nm, or 8 pixels, from the nucleus edge) (Kind et al., 2013).

STORM original data was processed by Insight3, ImageJ, and finally reconstructed to an image by home-written MATLAB scripts (Rust et al., 2006).

The number of n showed in the figure legends means the cumulative number from repeated experiments.

### Western blot

The cell lysates were blotted against the following primary antibodies: lamin B1 (sc6216, Santa Cruz, dilution 1:500) and β-actin (sc47778, Santa Cruz, dilution 1:500). The blots were visualized with peroxidase-coupled secondary antibodies.

### PI staining

Cells grown on 60 mm dish were digested by trypsin and collected to 1.5 mL tube. After being washed with PBS twice, cells were fixed in pre-chilled 75% ethanol at −20 °C overnight. The fixed cells were washed to remove ethanol, and then incubated in solution of 100 µg/mL RNase A and 0.2% Triton X-100 for 30 min at 37 °C. Subsequent centrifugation of the samples was followed by a wash in PBS and staining with PI solution (50 µg/mL PI, 0.2% Triton X-100) at room temperature for 30 min. Cells stained with PI were analyzed in Flow cytometer (BD LSRFortessa^TM^) directly (Krishan, 1975).

### Chromosome painting

Cells were grown on 22 × 22 mm^2^ coverslips. After the coverage of cells reached 70%–80%, cells were fixed at −20 °C for 20 min in a pre-chilled solution of methanol and acetic acid at 3:1 ratio and then treated with 10 µL of probe mix with 5 µL of each probe. The probe mix immersed cells were covered with a glass slide (25 × 75 mm) and sealed with rubber cement. The sample and probe were denatured simultaneously by heating slide on a hotplate at 75 °C for 2 min and incubated in a humidified chamber at 37 °C overnight. The coverslip was removed carefully from slide, washed in 0.4× SSC at 72 °C for 2 min, and then in 2× SSC, 0.05% Tween-20 at room temperature for 30 seconds. The labeled cells were rinsed briefly in PBS and finally mounted with ProLong® Diamond Antifade Mountant with DAPI (P36962, Thermo Fisher Scientific).

Chromosome 2 painting probe mix was XCP 2 green (D-0302-100-FI XCP 2, Metasystems), chromosome 18 painting probe mix was XCP 18 orange (D-0318-100-OR XCP 18, Metasystems) and chromosome 19 painting probe mix was XCP 19 orange (D-0319-100-OR XCP 19, Metasystems).

### ChIP-seq

ChIP-seq experiments were processed as previously described (Ai et al., 2017) from cultured MBA-MD-231 cells. The cells were collected and fixed with 1% formaldehyde (Sigma) at room temperature for 3 min, followed by 125 mmol/L glycine (Amresco) quenching. About 0.8 mol/L fixed cells for each replicate were resuspended with 1 mL hypotonic buffer (20 mmol/L Hepes pH 7.9, 10 mmol/L KCl, 1 mmol/L EDTA, 10% glycerol, 0.2% NP-40) and incubated in ice for 20 min. After centrifugation at 3,000 rpm for 5 min at 4 °C, the supernatant was discarded and the nuclei pellets were resuspended with 30 μL nuclei lysis buffer (1% SDS, 10 mmol/L EDTA, 50 mmol/L Tris-HCl pH 8.0). After lysis at 4 °C for 30 min, add 70 μL dilution buffer (0.01% SDS, 1% Triton X-100, 2 mmol/L EDTA, 20 mmol/L Tris-HCl pH 8.0, 200 mmol/L NaCl) to totally 100 μL with 0.3% SDS. The sample is transfered to 200 μL PCR tubes at this step. Chromatin was sheared into mainly 200–800 bp fragments with Q800R sonicator, at 80% power for 4 min, with 15 s ON and 30 s OFF. After sonication, the Triton X-100 in system was added to finally 1%. The chromatin was centrifuged at 20,000×*g* for 15 min at 4 °C. The supernatant was saved to new PCR tubes and precleared with 8 μL protein A dynabeads (Invitrogen) at 4 °C for 1 h, followed by adding lamin A antibody (ab26300, Abcam). About 1/50 of chromatin was taken out as the input. The chromatin-antibody mixture was incubated overnight at 4 °C. Each 15 μL Protein A dynabeads blocked by 1% BSA/PBS were added into chromatin-antibody mixture and rotated together for 4 h at 4 °C. Beads were washed with 150 μL low salt buffer for 3 min × 3 times, then for 1 min with 150 μL high salt buffer, and finally transfered into new tubes with 150 μL low salt buffer. Then ChIP mentation method was applied (Akhtar et al., 2019) to add adaptors to ChIPed fragments and input fragments. After pheno-chloroform purification, the adaptor-ligated fragments were amplified by Q5 polymerase (NEB) to make libraries with Illumina Nextera index primers. Cycles were determined in advance. ChIPed groups were amplified for 10 cycles and input groups were amplified for 8 cycles. After size selection for 200–1000 bp fragments, the libraries were quantified with Qubit for concentrations and sequenced with paired-end 150 bp reads on Nova 6000 platform (Illumina).

### Hi-C experiment

Hi-C experiment was performed following the *in situ* Hi-C protocol (Rao et al., 2014). Briefly, cells were grown to about 70%–80 % confluence, washed with PBS, crosslinked with 1%, *v*/*v* formaldehyde solution, and the reaction was quenched by 0.2 mol/L glycine solution. Cells were lysed and DNA was then cut with MboI and the overhangs were filled with a biotinylated base. Free ends were then ligated together *in situ*. Crosslinks were reversed, the DNA was sheared to 300–500 bp and then biotinylated ligation junctions were recovered with streptavidin beads.

Sequencing libraries were generated using standard Illumina library construction protocol. Briefly, ends of sheared DNA were repaired and the blunt ends were added an “A” base to ligate with Illumina’s adapters that have a single ‘T’ base overhang. Then DNA was PCR amplified for 8–12 cycles. At last, products were purified using AMPure XP system and sequenced through XTen (Illumina).

### ChIP-seq data analysis

Sequencing reads were aligned to hg19 reference genome using Bowtie2 with default parameters (Langmead and Salzberg, 2012). Duplicate reads were removed using Picard’s Mark Duplicate algorithm (Brakemann et al., 2011). LADs were called using enriched domain detector (EDD) peak-calling algorithm with parameters of 11 kb bin size and a gap penalty of 5 (Lund et al., 2014).

### Hi-C data analysis

Hi-C data analysis was performed with HiC-Pro (Servant et al., 2015). Briefly, reads were first aligned on the hg19 reference genome. Uniquely mapped reads were assigned to restriction fragments. Then the invalid ligation products were filtered out, and eligible read pairs were counted to build Hi-C contact maps. Hi-C raw matrices were normalized using ICE method (Imakaev et al., 2012) which assumes that all genomic loci have equal visibility and applies iterative biases correction to each sample. After normalization, we obtained genome-wide relative contact maps that are comparable between samples. All subsequent analysis, such as inter-chromosomal interaction analysis, A/B compartment and TAD calling, were based on the normalized interaction matrices. To compare the interaction frequency between samples, contact matrices were normalized by the number of total valid interactions of each sample.

Compartment A/B analysis. ICE-normalized 500-kb resolution matrices were used to detect chromatin compartments by R package HiTC (Servant et al., 2012). The whole genome was divided into two compartments based on the positive or negative values of the first principal component. The part with higher gene density was assigned as compartment A and the other part as compartment B. To calculate the inter-compartment (AB) interactions and intra-compartment (AA + BB) interactions, contacts within 2 Mb were removed because they were regarded as interactions within TADs.

TAD analysis. ICE-normalized 40-kb resolution matrices were used to detect TAD by Perl script matrix2insulation.pl (http://github.com/blajoie/crane-nature-2015). Insulation scores were calculated for each 40-kb bin, and the valleys of insulation score curves were defined as TAD boundaries. TADs smaller than 200 kb were filtered out as in previous method (Crane et al., 2015).

### RNA-seq data analysis

First, all sequencing reads were mapped to human reference genome hg19 by Hisat2 (Kim et al., 2015) with default parameters, and then counted for each gene by summarize Overlaps function (Lawrence et al., 2013) in Bioconductor. Then, DESeq2 (Love et al., 2014) was used to calculate differentially expressed genes (upregulated: fold change > 2, FDR < 0.05; downregulated: fold change < 0.5, FDR < 0.05). GO enrichment analysis was performed using Enrichr (Kuleshov et al., 2016).

## Electronic supplementary material

Below is the link to the electronic supplementary material.

Supplementary material 1 (PDF 436 kb)

Supplementary material 1 (PDF 1959 kb)

Supplementary material 1 (ZIP 610 kb)
