# Supplementary material for: Nuclear peripheral chromatin-lamin B1 interaction is required for global integrity of chromatin architecture and dynamics in human cells
Source: Protein Cell. 2020 Nov 5;13(4):258–80. doi: 10.1007/s13238-020-00794-8 (PMC8934373; doi:10.1007/s13238-020-00794-8)
Supplement: Supplementary file 1 — Supplementary material 1 (PDF 436 kb) [file 13238_2020_794_MOESM1_ESM.pdf]

Supplementary materials

Table S1 Quality control statistics for lamin A ChIP-seq data processing.

	WT-r1	WT-r2	WT-r3	WT-input
**Raw Reads**	13,282,558	13,159,405	16,918,646	21,972,529
**Cut adapter**	13,278,333	13,155,291	16,913,689	21,969,306
**Mapping Rate**	97.11%	97.30%	96.97%	96.45%
**rm duplicates**	11,437,609	11,339,059	14,303,429	18,954,709
**rm LowMAPQ**	10,209,282	10,170,347	12,685,977	15,976,666
	**KO-r1**	**KO-r2**	**KO-r3**	**KO-input**
**Raw Reads**	16,354,928	11,908,170	17,430,764	20,086,104
**Cut adapter**	16,349,893	11,903,514	17,425,796	20,082,833
**Mapping Rate**	96.87%	96.11%	96.94%	96.39%
**rm duplicates**	13,957,698	10,481,812	14,659,780	17,239,813
**rm LowMAPQ**	12,330,442	9,163,410	12,946,388	14,833,466

Remaining read pairs after each filtering step.

Table S2 LAD coverage on each chromosome in WT and lamin B1-KO cells.

Chromosome	WT (%)	Lamin B1 KO (%)
**chr1**	41	26
**chr2**	51	34
**chr3**	63	35
**chr4**	76	35
**chr5**	58	34
**chr6**	49	25
**chr7**	46	33
**chr8**	53	36
**chr9**	37	22
**chr10**	33	31
**chr11**	52	35
**chr12**	46	30
**chr13**	50	21
**chr14**	37	23
**chr15**	21	23
**chr16**	20	30
**chr17**	11	13
**chr18**	53	37
**chr19**	1	9
**chr20**	26	26
**chr21**	33	15
**chr22**	0	5
**chrX**	66	48

Table S3 Diagram of different classes of LADs.

LAD (1) or non-LAD (0)

	**Constitutive**		**Facultative**	
	cLAD	ciLAD	fLAD	fiLAD
**MDA-MB-231**	1	0	1	0
**hESC**	1	0		
**HT1080**	1	0	<=2	>=1
**Tig3**	1	0		
**Total size (Mb)**	612	1011	774	466

Table S4 Quality control statistics for Hi-C data processing.

	**WT-r1**	**WT-r2**	**Lamin B1 KO-r1**	**Lamin B1 KO-r2**
**Total read pairs[1]**	138,193,618	223,885,207	250,362,318	253,024,668
**Uniquely aligned read pairs[2]**	108,155,179	172,561,205	182,642,642	194,536,722
	78.26%	77.08%	72.95%	76.88%
**Valid interaction[3]**	95,264,155	152,469,233	148,123,567	166,884,723
	88.08%	88.36%	81.10%	85.79%
**Self-Circle[4]**	123,564	178,371	520,046	222,818
	0.11%	0.10%	0.28%	0.11%
**Dangling-end[5]**	2,726,851	4,213,273	13,818,685	6,521,973
	2.52%	2.44%	7.57%	3.35%
**Valid interaction rmdup[6]**	79,041,927	131,026,015	129,693,005	134,947,244
	73.08%	75.93%	71.01%	69.37%
**Trans_interaction[7]**	7,834,781	13,603,317	15,747,086	16,307,003
	9.91%	10.38%	12.14%	12.08%
**Cis_interaction[8]**	71,207,146	117,422,698	113,945,919	118,640,241
	90.09%	89.62%	87.86%	87.92%
**Cis_shortRange[9]**	22,113,855	34,770,742	37,869,246	39,050,597
	31.06%	29.61%	33.23%	32.92%
**Cis_longRange[10]**	49,093,291	82,651,956	76,076,673	79,589,644
	68.94%	70.39%	66.77%	67.08%

The percentage denominators of [2] are the read pair numbers in [1]; the percentage denominators of [3][4][5][6] are the uniquely aligned read pair numbers in [2]; the percentage denominators of [7][8] are in [6]; and the percentage denominators of [9][10] are in [8].

Table S5 Design of sgRNAs

Genome coordinates	Sequence of sgRNAs	Compartment		LAD		Repeat number
		WT	KO	WT	KO	
Chr2: 1218818-1227201	GAGGUGCUGGAUCGCUGUAGAGG	B	B	Yes	Yes	100
Chr2: 6378242-6379050	AGCGUCACCGUUCACUGCGGCGG/GGG	B	B	Yes	Yes	23
Chr2: 36410123-36411725	AUAUCUAUCUAUCGAUACAUAGG/GGG<br>GUAUAUAUCUAUAUCUAUCUAUGG	B	B	Yes	Yes	24
Chr2: 114408189-114411271	GAUACCCUGAGCUCAUGACUCAGAGG	NA	NA	No	No	40
Chr2: 135364479-135367491	GACAGACCCCGAGAACUGUGCUGG	B	B	No	Yes	60
Chr2: 235554023-235558288	GCAGUGGGAGUAGGGAGGCAGUGGG	A	A	No	No	65
Chr18: 13673058-13676522	GCUCAGGAAUGUUAAUAAUCAGG	B	A	No	No	63
Chr18: 44926826-44929361	GUGGAAAGGCACAGUGUGGUGG	A	A	No	No	23
Chr18: 77567735-77569657	GGGCCCAUAACGUGGAGUGUGG	A	A	No	No	27
Chr19: 1627737-1629139	GAAGGGGACAGCAGAGCUCACGGG/AGG	A	A	No	No	36
Chr19: 59050388-59054262	CUGUCUGAGGAGGGAAGCAGGG	A	A	No	No	69

Firstly, the human genome sequence of chromosome 2 (GRCh37/hg19 Assembly) was downloaded from the UCSC genome browser. Secondly, the undetermined regions “Ns” were replaced by randomly generated nucleotides “A”, “T”, “G”, or “C”. Thirdly, the modified sequence was inputted to the Tandem Repeat Finder bioinformatics tool to identify tandem repeats. Lastly, the results were summarized. Highly conserved repeats with less mutation and proper repeat unit length and repeat number were selected as candidates for live cell imaging.

Table S6 Statistics of LAD status alterations in WT and lamin B1 KO cells

	non-LAD	LAD	Non-LAD to LAD	LAD to non-LAD
**Total size (Mb)**	1,401	821	74	565
**Gene #**	20,142	2,878	508	2,768
**Gene density (#/Mb)**	14.38	3.51	6.86	4.90
**Upregulated gene #**	336	11	1	11
**Downregulated gene #**	399	8	4	82
