# Supplementary material for: Nuclear peripheral chromatin-lamin B1 interaction is required for global integrity of chromatin architecture and dynamics in human cells
Source: Protein Cell. 2020 Nov 5;13(4):258–80. doi: 10.1007/s13238-020-00794-8 (PMC8934373; doi:10.1007/s13238-020-00794-8)
Supplement: Supplementary file 2 — Supplementary material 1 (PDF 1959 kb) [file 13238_2020_794_MOESM2_ESM.pdf]

Supplementary materials

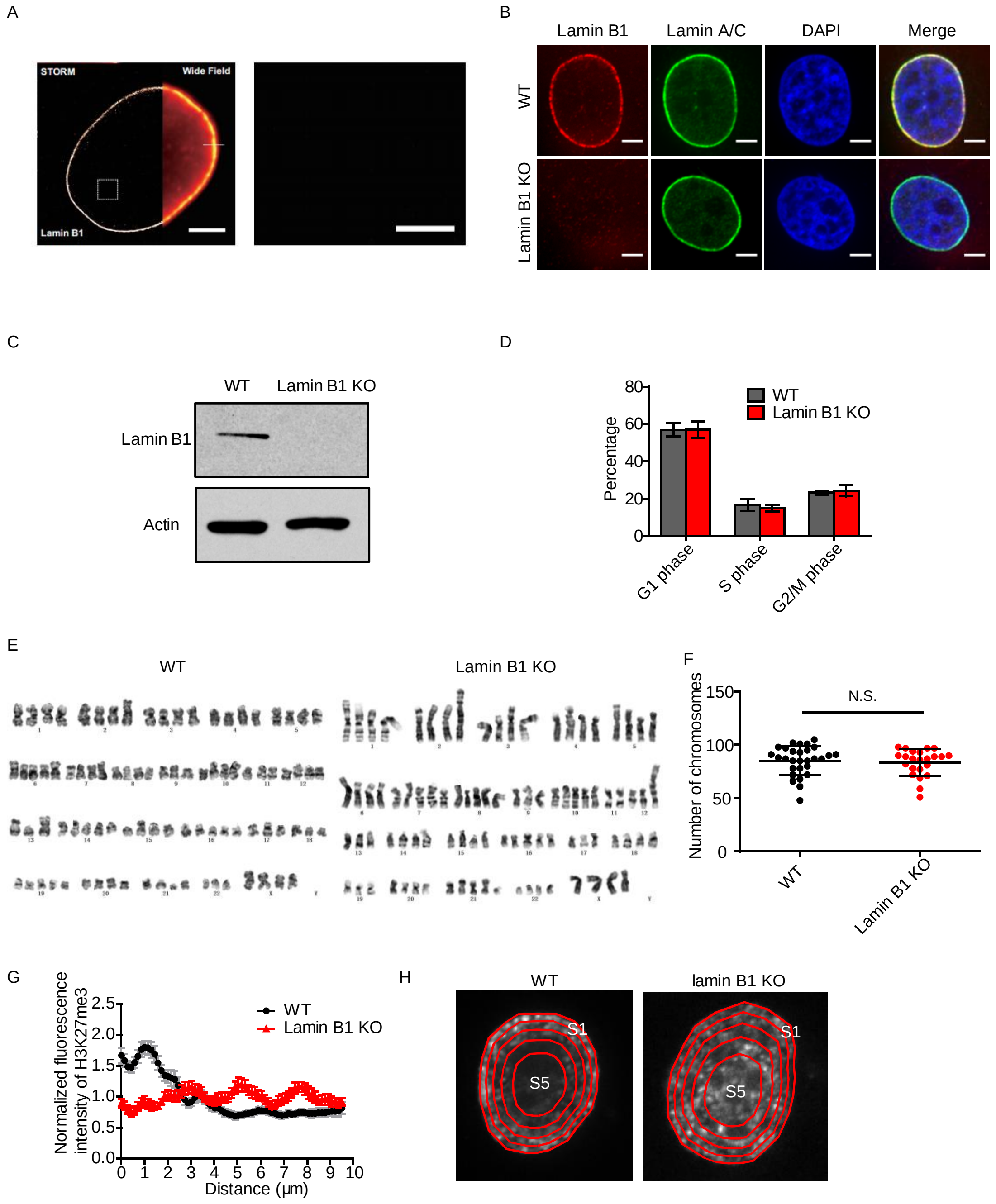

Fig. S1 Preparation of lamin B1 KO cell lines.

(A) STORM image and wide-field image of lamin B1 in MDA-MB-231 cells. Scale bar: 5 μm. Magnified image of the boxed area shows that lamin B1 almost has no presence in nucleoplasm. Scale bar: 1 μm.

(B) Immunofluorescence of the chosen lamin B1-KO clone with lamin B1 and lamin A/C antibody. WT MDA-MB-231 cell line is used as positive control. The result indicates that lamin B1 is totally knocked out in the chosen KO clone, while lamin A/C

is unaffected. The lamin B1 images are shown under the same intensity threshold between WT and lamin B1-KO cells.

(C) Western blot of the chosen lamin B1 KO clone with lamin B1 antibody. Actin is used as loading control. WT MDA-MB-231 cell line functions as positive control.

(D) Percentage of cells in G1, S, and G2/M phase in 3 independent experiments.

(E) A representative karyotyping analysis of the WT and lamin B1-KO cell lines used in this study.

(F) The statistics of chromosome number in WT and lamin B1-KO cells. Unpaired *t*-test.

(G) Averaged fluorescence intensity profiles along the nuclear diameter in WT (*n*=27) and lamin B1-KO (*n*=29) cells immunostained with antibodies against H3K27me3.

(H) Each nucleus is divided into 5 shells with equal area form the nuclear periphery to interior.

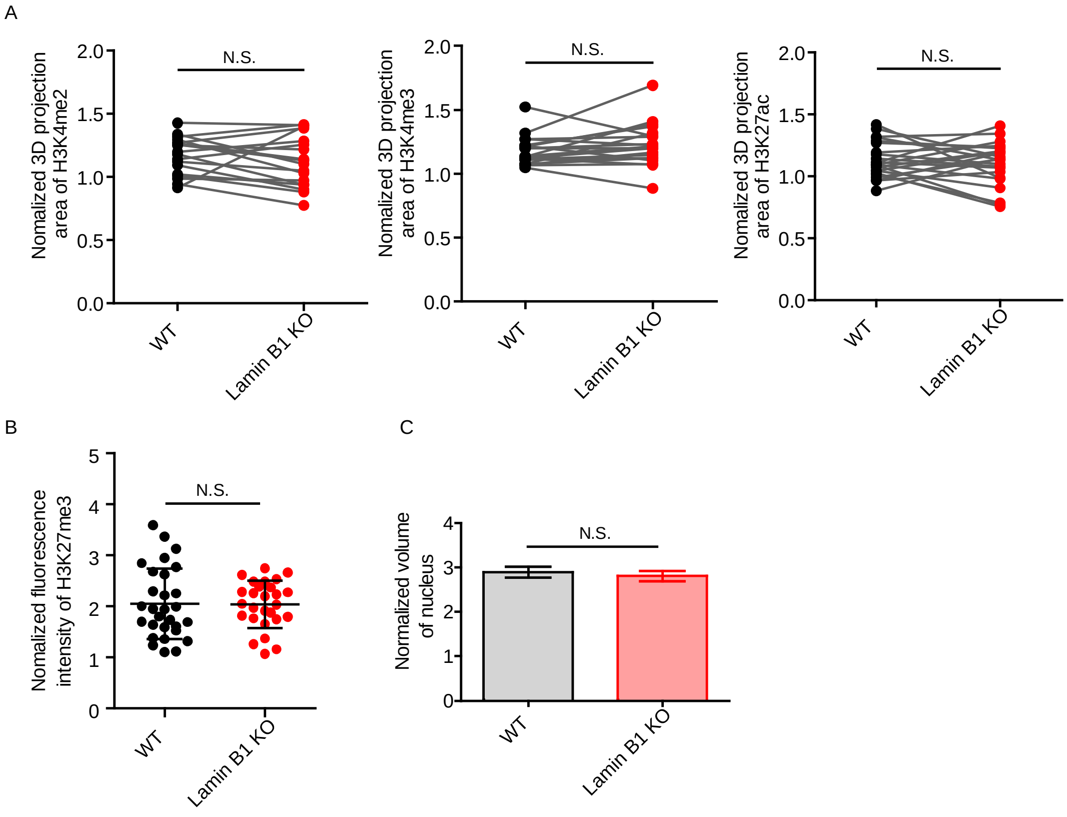

Fig. S2 Quantification of lamin B1 KO cells.

(A) Normalized 3D projection nuclear area of the counted cells in Fig. 1D. Statistical analysis shows nuclear area stays consistent in WT and lamin B1-KO cells. Paired *t*-test. 2 independent experiments.

(B) Normalized total fluorescence intensity of immunostaining signals of H3K27me3. Statistical analysis shows the level of H3K27me3 stays consistent. Unpaired *t*-test. 2 independent experiments.

(C) Quantification of nuclear volume based on DAPI staining in WT (*n*=18) and lamin B1-KO (*n*=16) cells. Mean ± SE.

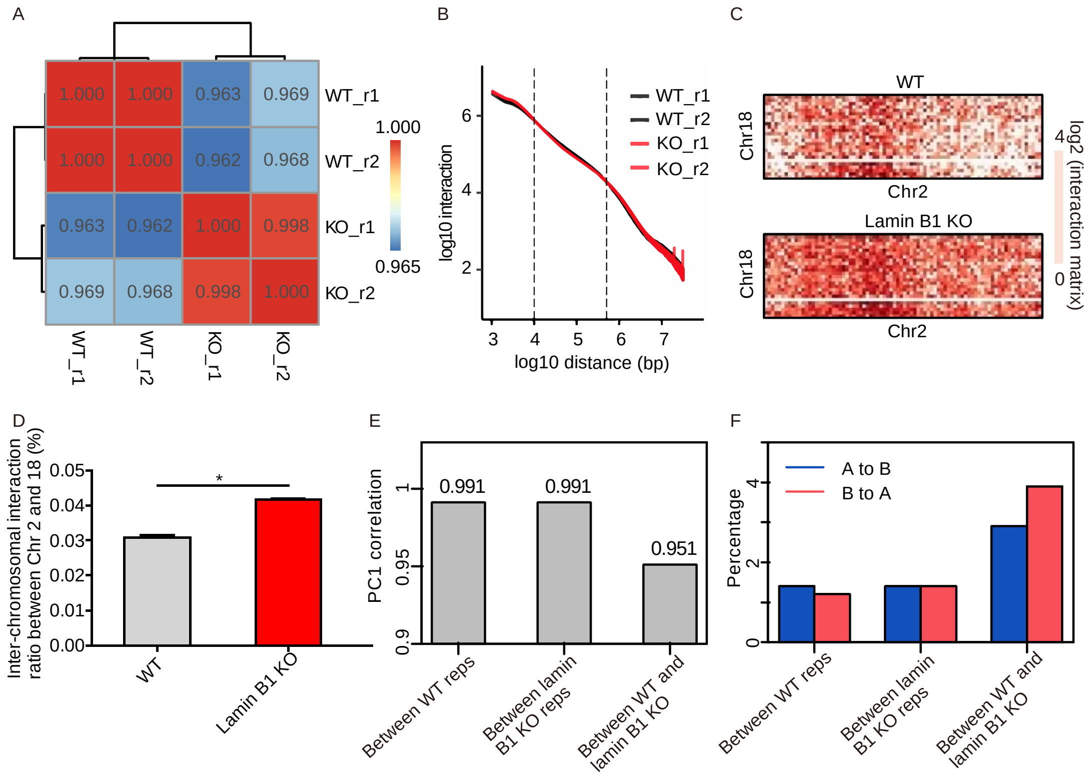

Fig. S3 Reproducibility analysis of Hi-C data.

(A) Pearson correlation coefficients of the whole genome interaction matrices (resolution: 500 kb) of WT and lamin B1-KO replicates.

(B) Hi-C interaction frequency as a function of genomic linear distance for WT and lamin B1-KO replicates.

(C) Normalized Hi-C trans-interaction matrices for chromosome 2 and 18 in WT and lamin B1-KO samples.

(D) Trans-interaction ratios of chromosome 2 and 18 in 2 WT replicates and lamin B1-KO replicates. Interaction numbers of chromosome 2 and 18 are normalized by the total interactions of the whole genome in each sample. * $P<0.05$, *t*-test.

(E) Pearson correlation coefficients of the whole genome PC1 values between WT replicates (r=0.991), lamin B1-KO replicates (r=0.991), WT and lamin B1-KO samples (r=0.951).

(F) Percentage of the whole genome A/B compartment transition between WT replicates, lamin B1-KO replicates, WT and lamin B1-KO samples.

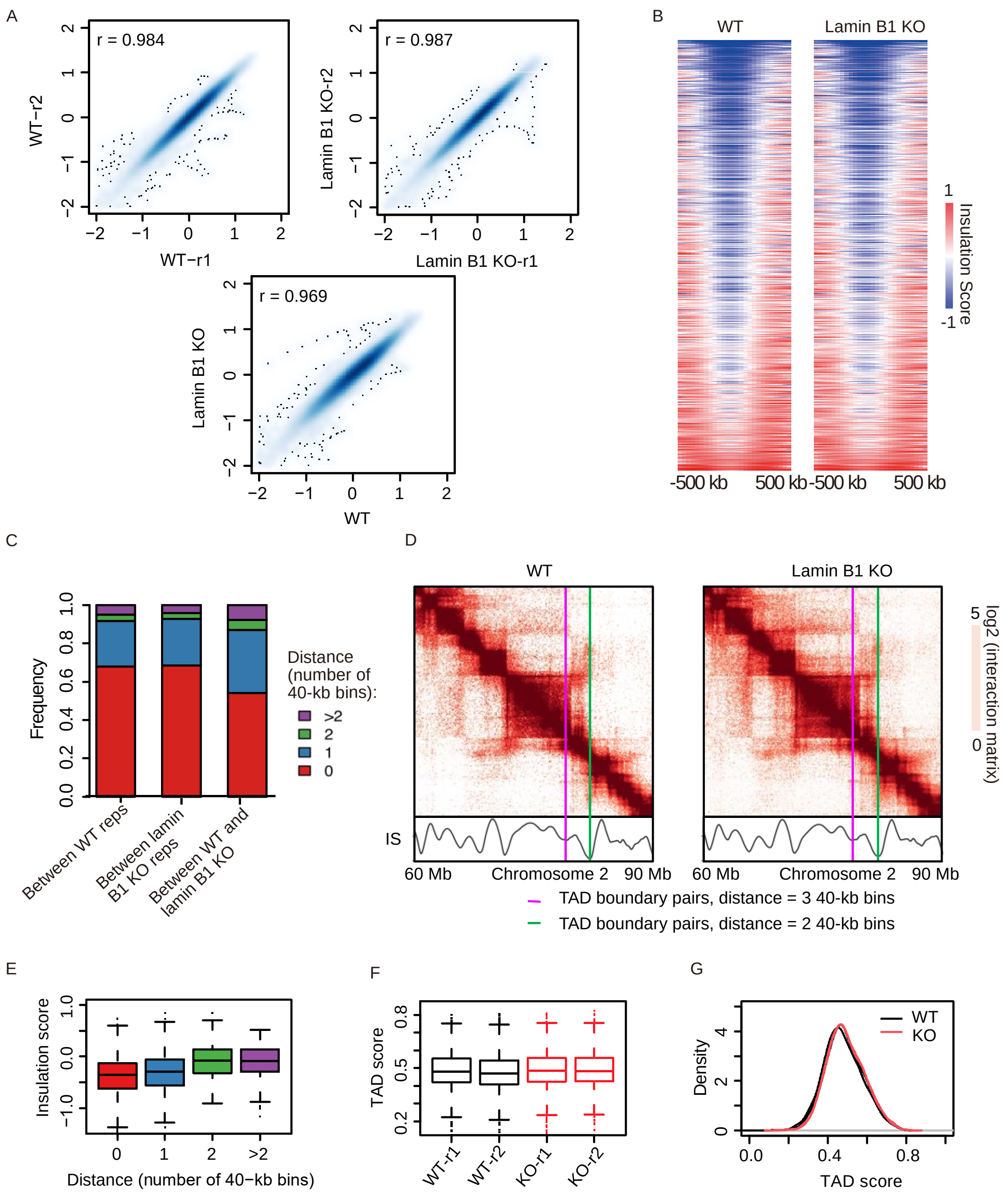

Fig. S4 TAD analysis

(A) Scatter plots of the whole genome insulation scores of WT replicates (Pearson correlation coefficient *r*=0.984), lamin B1-KO replicates (Pearson correlation coefficient *r*=0.987), WT and lamin B1-KO samples (Pearson correlation coefficient *r*=0.969).

(B) Heatmaps of insulation score around TAD boundaries in WT and lamin B1-KO cells. Heatmaps are organized according to the sum of insulation score around each boundary (±500 kb).

(C) Histogram of distance frequency of the most adjacent TAD boundary pairs between WT and lamin B1-KO cells, as well as between replicates of WT and lamin B1-KO-cells (40 kb bin).

(D) Example of two most adjacent TAD boundary pairs in WT and lamin B1 KO-cells, with the distance of 3 (purple) and 2 (green) 40-kb bins.

(E) Boxplot of mean insulation scores of most adjacent TAD boundary pairs in WT and lamin B1-KO cells at a distance of indicated numbers. Together with Fig. S3D, the insulation score valleys at identical boundary pairs are lower and sharper than the changed ones, indicating that shifted TAD boundaries are due to variance upon calculation.

(F) Boxplot of TAD scores of WT and lamin B1-KO replicates. TAD score=intra-TAD interactions / (intra- + inter-TAD interactions).

(G) TAD score distribution of WT and lamin B1-KO samples.

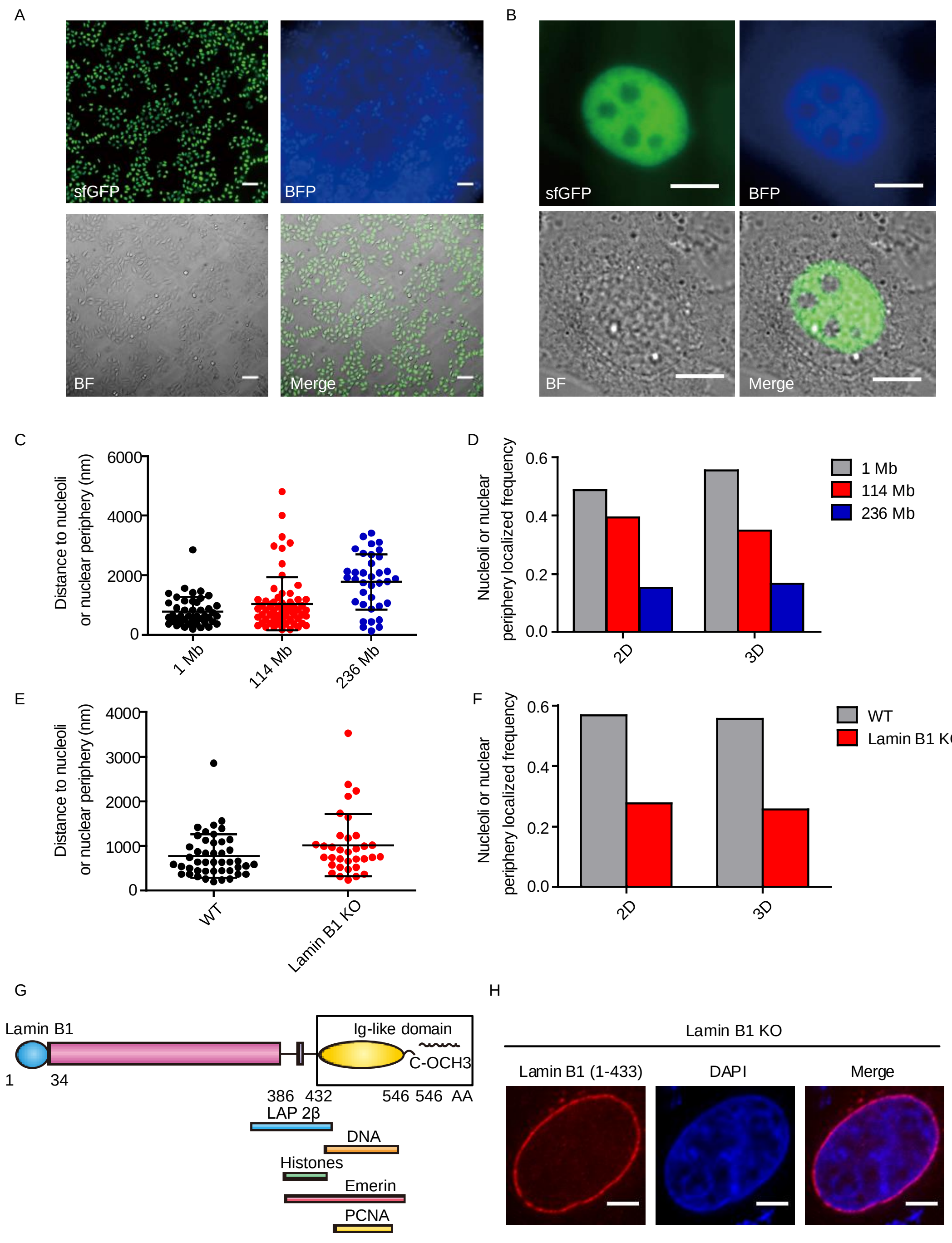

Fig. S5 Construction of SunTag stable cell line, comparison between 2D and 3D images and description of lamin B1 truncation.

(A) Fluorescence images of the SunTag cell line under 10× magnification with Dox induction. BFP and bright field (BF) are also shown. All cells display similar expression level of both dCas9-(GCN4)×24 and scFv-GCN4-sfGFP. Scale bars: 100 μm.

(B) Fluorescence images of the SunTag cell line under 100× magnification with Dox induction. BFP and BF are also shown. The absence of fluorescent signal of both blue and green channel in nucleoli indicates that this method is superior to the conventional dCas9-GFP labeling method which shows severe nucleoli aggregation. Scale bars: 10 μm.

(C) Quantification of distance from three genomic loci on chromosome 2 to nucleoli or nuclear periphery in reconstructed 3D images. 2 independent experiments.

(D) Nucleoli and nuclear periphery localized frequency of 3 genomic loci on chromosome 2 from 2D and 3D images. Each locus is assigned to this localization according to the rule that the minimum distance to nucleoli or nuclear envelope is less than 4 pixels (~ 640 nm).

(E) Quantification of distance from 1 Mb locus on chromosome 2 to nucleoli or nuclear periphery in WT and lamin B1-KO cells in reconstructed 3D images. 2 independent experiments.

(F) Nucleoli and nuclear periphery localized frequency of 1 Mb locus on chromosome 2 from 2D and 3D images. Each locus is assigned to this localization according to the rule that the minimum distance to nucleoli or nuclear envelope is less than 4 pixels (~ 640 nm).

(G) Diagram of lamin B1 and binding sites of interaction partners. The Ig-like domain of lamin B1 mediates direct and indirect interaction between lamin B1 and chromatin through DNA and other proteins.

(H) Lamin B1 (1-433) truncation without Ig-like domain localizes at the nuclear periphery when expressed in lamin B1-KO cells. Red: Lamin B1 (1-433) truncation fused with mCherry. Blue: DAPI staining. Scale bars, 5 μm.

A

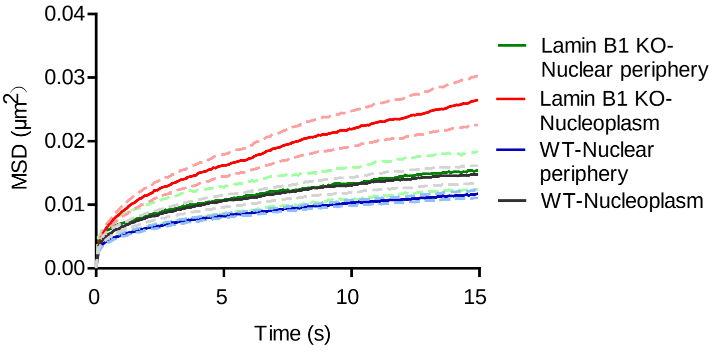

B

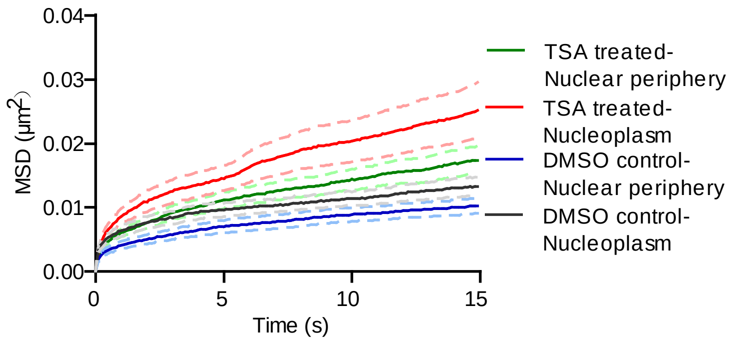

Fig. S6 Dynamic changes of 1Mb loci on chromosome 2 at the nuclear periphery and nucleoplasm.

(A) MSD curves of 1Mb loci on chromosome 2 localized at the nuclear periphery and nucleoplasm of WT and lamin B1-KO cells, separately. Mean ± SE. Depletion of lamin B1 increases 1Mb loci dynamics in both periphery and nucleoplasm. 3 independent experiments.

(B) MSD curves of 1Mb loci on chromosome 2 localized at the nuclear periphery and nucleoplasm of DMSO-treated control and TSA-treated cells, separately. Mean ± SE. TSA treatment increases 1Mb loci dynamics in both nuclear periphery and nucleoplasm. 3 independent experiments.

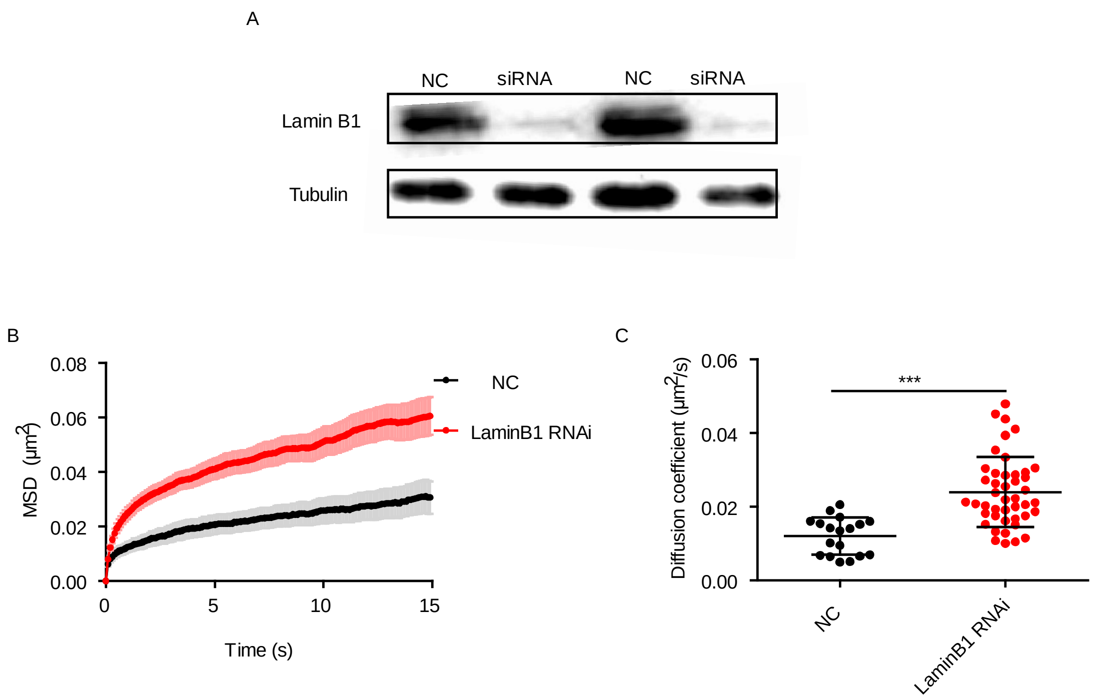

Fig. S7 Knock-down of lamin B1 increased the dynamics of chromatin

(A) Western blot showing decreased levels of *LMNB1* in 2 independent RNAi experiments. Tubulin provided a loading control.

(B) MSD curves of 1Mb loci on chromosome 2 in negative control (NC) (*n*=18) and lamin B1 knock-down (*n*=44) cells. Mean ± SE.

(C) The diffusion coefficient of 1Mb loci in NC (*n*=18) and lamin B1 knock-down (*n*=44) cells. Mean ± SD. *** $P < 0.001$, Mann–Whitney test. 2 independent experiments.

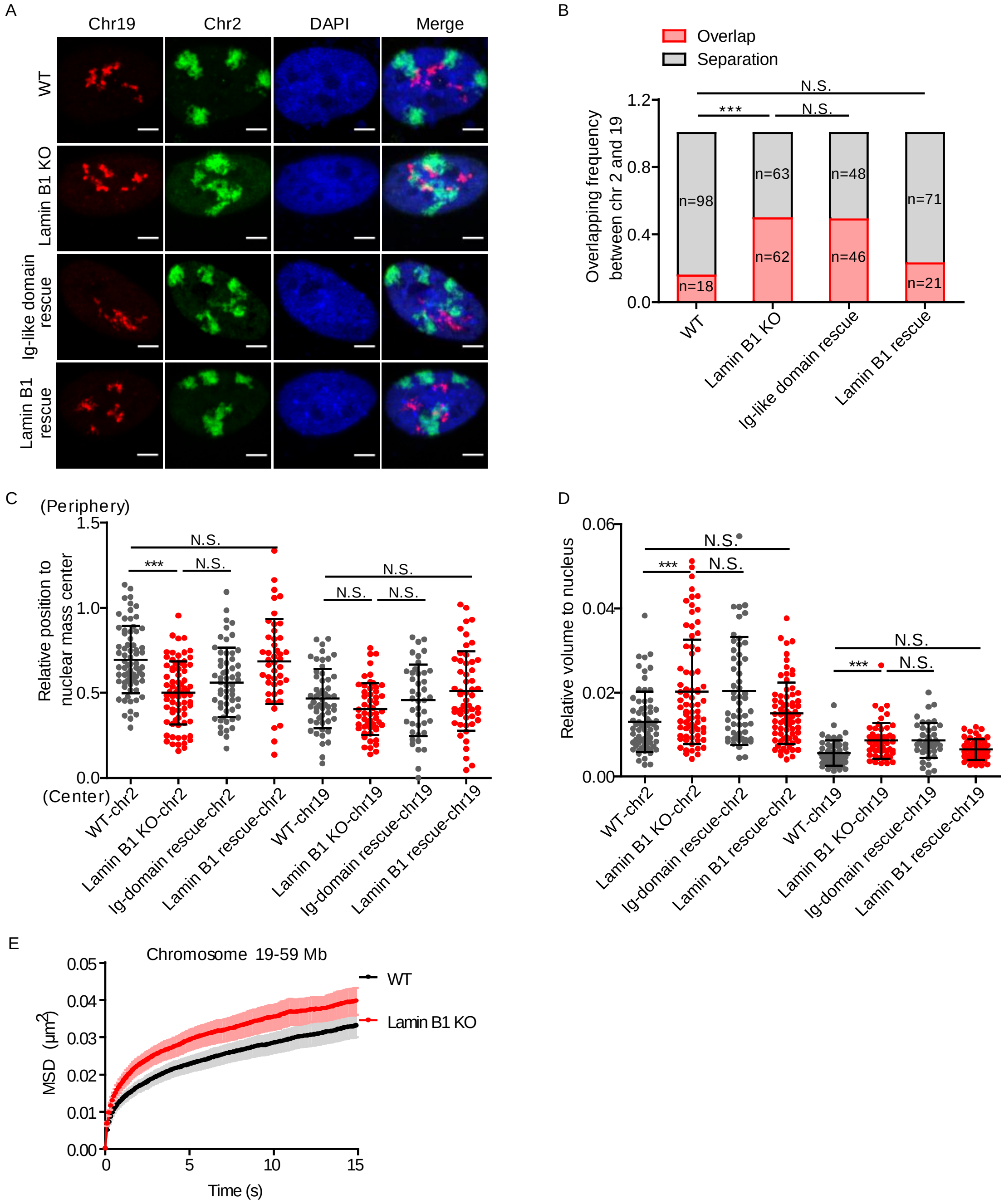

Fig. S8 Phenotypes of chromosome 19 upon lamin B1 depletion

(A) Representative 3D-projection chromosome painting images of chromosome 2 and 19. Green: FISH signal of chromosome 2. Red: FISH signal of chromosome 19. Blue: DAPI staining. The maximum intensity projections of nuclear Z stacks are displayed. Scale bars, 5 μm.

(B) Quantification of the overlap frequency between chromosome 2 and chromosome 19 territories. The ratio of cells presenting territory interaction between chromosome 2

and chromosome 19 in WT cells is significantly smaller than that in lamin B1-KO cells. *** $P < 0.001$, Fisher's exact test. 2 independent experiments.

(C) Quantification of the nuclear localization of chromosomes based on their relative distances from the chromosome mass center to the nuclear mass center. This distance is normalized by the cubic root of the nuclear volume. Mean ± SD. *** $P < 0.001$, Mann–Whitney test. 2 independent experiments.

(D) Quantification of the volumes occupied by chromosome 2 and 19 relative to the nuclear volume. Chromosomes in lamin B1-KO cells show significantly larger relative volumes than WT cells. Mean ± SD. *** $P < 0.001$, Mann–Whitney test. 2 independent experiments.

(E) MSD curves of 59 Mb loci on chromosome 19 in WT (n=30) and lamin B1-KO (n=30) cells. Mean ± SE. 3 independent experiments.

A

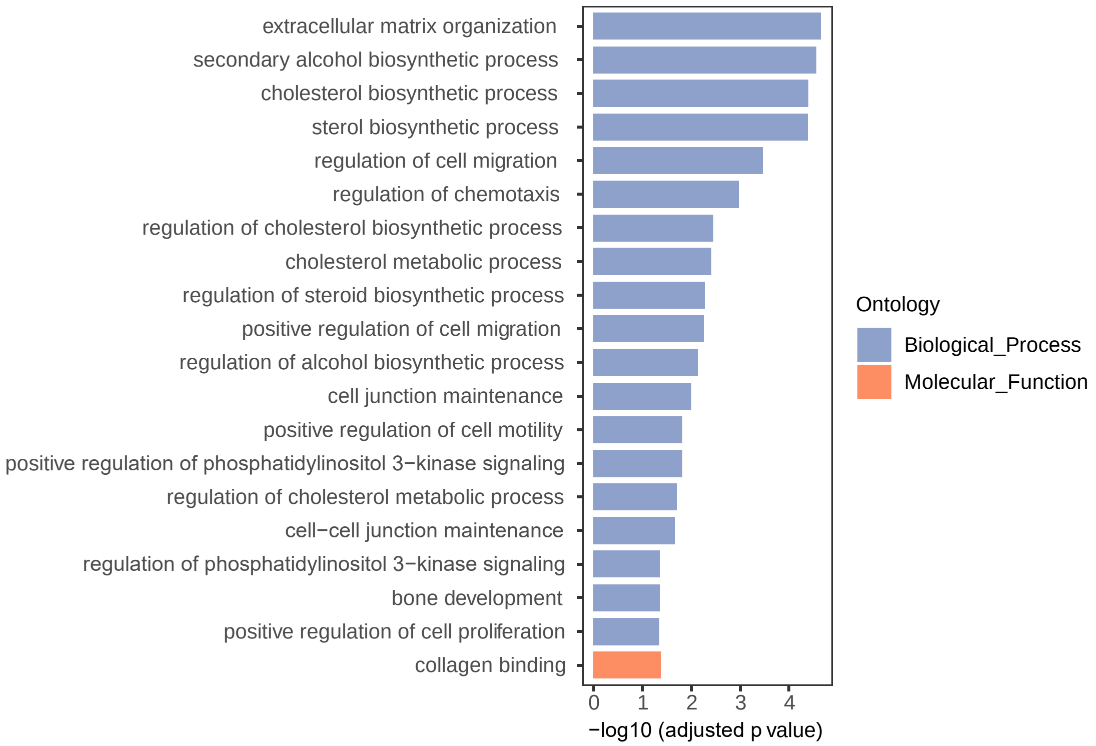

B

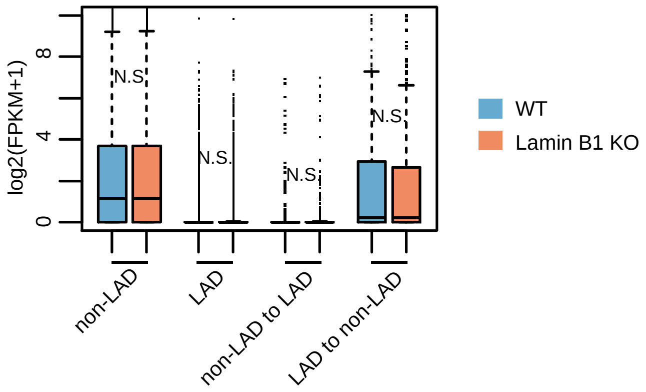

Fig. S9 RNA-seq analysis

(A) GO analysis of down-regulated genes upon lamin B1 depletion.
(B) Expression levels in regions with four types of LAD status. Non-LAD are regions that are non-LAD in both WT and lamin B1 KO cells, and LAD are regions that are LAD in both WT and lamin B1 KO cells. Unpaired *t*-test.
